# Characterizing the Key Metabolic Pathways of the Neonatal Mouse Heart Using a Quantitative Combinatorial Omics Approach

**DOI:** 10.3389/fphys.2018.00365

**Published:** 2018-04-11

**Authors:** Maciej M. Lalowski, Susann Björk, Piet Finckenberg, Rabah Soliymani, Miikka Tarkia, Giulio Calza, Daria Blokhina, Sari Tulokas, Matti Kankainen, Päivi Lakkisto, Marc Baumann, Esko Kankuri, Eero Mervaala

**Affiliations:** ^1^Department of Biochemistry, Department of Developmental Biology, Faculty of Medicine, Helsinki Institute of Life Science (HiLIFE) and Medicum, Meilahti Clinical Proteomics Core Facility, University of Helsinki, Helsinki, Finland; ^2^Medicum, Department of Pharmacology, Faculty of Medicine, PB63, University of Helsinki, Helsinki, Finland; ^3^Institute for Molecular Medicine Finland (FIMM), Helsinki Institute of Life Science, University of Helsinki, Helsinki, Finland; ^4^Medical and Clinical Genetics, University of Helsinki and Helsinki University Hospital, Helsinki, Finland; ^5^Medicum, Department of Clinical Chemistry and Hematology, Faculty of Medicine, PB63, University of Helsinki, Helsinki, Finland

**Keywords:** neonatal heart, omics, fructolysis, hypoxia, cardiomyocyte proliferation, regeneration

## Abstract

The heart of a newborn mouse has an exceptional capacity to regenerate from myocardial injury that is lost within the first week of its life. In order to elucidate the molecular mechanisms taking place in the mouse heart during this critical period we applied an untargeted combinatory multiomics approach using large-scale mass spectrometry-based quantitative proteomics, metabolomics and mRNA sequencing on hearts from 1-day-old and 7-day-old mice. As a result, we quantified 1.937 proteins (366 differentially expressed), 612 metabolites (263 differentially regulated) and revealed 2.586 differentially expressed gene loci (2.175 annotated genes). The analyses pinpointed the fructose-induced glycolysis-pathway to be markedly active in 1-day-old neonatal mice. Integrated analysis of the data convincingly demonstrated cardiac metabolic reprogramming from glycolysis to oxidative phosphorylation in 7-days old mice, with increases of key enzymes and metabolites in fatty acid transport (acylcarnitines) and β-oxidation. An upsurge in the formation of reactive oxygen species and an increase in oxidative stress markers, e.g., lipid peroxidation, altered sphingolipid and plasmalogen metabolism were also evident in 7-days mice. *In vitro* maintenance of physiological fetal hypoxic conditions retained the proliferative capacity of cardiomyocytes isolated from newborn mice hearts. In summary, we provide here a holistic, multiomics view toward early postnatal changes associated with loss of a tissue regenerative capacity in the neonatal mouse heart. These results may provide insight into mechanisms of human cardiac diseases associated with tissue regenerative incapacity at the molecular level, and offer a prospect to discovery of novel therapeutic targets.

## Introduction

In the early postnatal days the mammalian heart needs to adapt to an increased workload and energy demand under increased blood oxygen levels (Patterson and Zhang, 2010). The heart cells rely on oxidative phosphorylation and versatile energy substrate dynamics, such as beta-oxidation of fatty acids, oxidation of lactate to pyruvate, and to a moderate extent glucose utilization, for production of adequate amounts of energy to compensate for the increased requirements. During the first week of postnatal heart development, major adaptive physiological transitions take place due to changes in oxygen pressure, workload and availability of substrates for energy metabolism, which induces a shift from glycolytic energy production to oxidative phosphorylation, as well as mitochondrial biogenesis and remodeling (Lopaschuk et al., 1994; Neary et al., 2014; Puente et al., 2014). The increase in postnatal environmental oxygen supply, with the subsequent up-regulation of oxidative metabolism, as well as a rise in production of mitochondrial reactive oxygen species (ROS), triggers cardiomyocytes to exit the cell-cycle shortly after birth (Puente et al., 2014; Nakada et al., 2017). The block of cytokinesis evokes binucleation of the cardiomyocytes, with the consequent loss of their proliferative capacity leading to terminal differentiation at 7 days postnatal (Soonpaa et al., 1996; Leu et al., 2001; Porrello and Olson, 2014). Subsequent heart growth is achieved through an increase in cell size, i.e., hypertrophy. The shift from fetal to adult metabolism during the first postpartum week has gained attention due to the observed loss of the heart's regenerative capacity at day 7 and after through adulthood (Porrello and Olson, 2014; Uygur and Lee, 2016; Vivien et al., 2016). An ischemic insult and myocardial infarction, for example, readily cause the death of billions of cardiomyocytes with substantial damage to the left ventricle and subsequent fibrotic scar formation, eventually leading to fatal arrhythmias and heart failure (Xin et al., 2013). In contrast to adult hearts, the newborn mouse heart possesses an intriguing inherent capacity to regenerate upon myocardial injury (Porrello and Olson, 2014; Uygur and Lee, 2016; Vivien et al., 2016). Elucidating the mechanisms associated with the immense repair potential and regenerative capacity early after birth can contribute to discovery of novel therapeutic targets for cardiac diseases causing cardiomyocyte loss and fibrosis that are significant contributors to cardiovascular mortality (Lerman et al., 2016).

The plausible basis of the ineffective cardiac regeneration in mammals is the low proliferative capacity of adult cardiomyocytes (Foglia and Poss, 2016). Since cardiomyocytes cannot undergo self-renewal, differentiation and repair, myocardial injury and cardiomyocyte loss remain untreatable, and novel strategies to increase cardiomyocyte renewal are explored (Sahara et al., 2015). In addition to neonatal mice, which are capable to regenerate their myocardial damage provoked by apical resection (Porrello et al., 2011) or left anterior descending artery ligation (Haubner et al., 2012) within 3 weeks, cardiomyocytes in the hearts of some vertebrate animal models e.g., newts and zebrafish (Poss et al., 2002; Witman et al., 2011) can de-differentiate in order to promote cardiomyocyte proliferation to compensate for lost cells after a myocardial infarction (MI). Furthermore, functional cardiac recovery of a human neonatal heart upon MI has been reported (Haubner et al., 2016). Further studies using neonatal mice as the model of cardiac regeneration have revealed that the new cardiomyocytes were generated from de-differentiation and proliferation of pre-existing mature cardiomyocytes (Jopling et al., 2010; Haubner et al., 2012; Mollova et al., 2013; Porrello et al., 2013; Senyo et al., 2013). The de-differentiated cardiomyocytes expressed markers characteristic of immature cardiomyocytes, had disassembled sarcomere structures and possessed an increased propensity for cell-cycle entry and thus proliferation (Jopling et al., 2010; Porrello et al., 2011), indicative of a connection between the cardiomyocyte contractile apparatus and the cell-cycle machinery (Vivien et al., 2016). However, the precise mechanisms underlying the regenerative capacity of the neonatal mouse model are only partially understood (Porrello et al., 2011; Haubner et al., 2012). Studies manipulating the cardiomyocyte genetic program or signaling environment to awaken innate regenerative programs have revealed that modulation of Neuregulin 1/v-erb-b2 avian erythroblastic leukemia viral oncogene homolog 4, NRG1-ERbB4 signaling (Bersell et al., 2009), Hippo-Yes associated protein, Hippo-YAP signaling (Heallen et al., 2013), cardiac innervation (Mahmoud et al., 2015; White et al., 2015), hypoxia and ROS (Puente et al., 2014), inflammatory response (Aurora et al., 2014) and miRNAs (Eulalio et al., 2012) play a key role in the regulation of cardiomyocyte proliferation and heart regeneration.

It is therefore clear that the regenerative capacity of the neonatal mouse model can be utilized as a valuable tool to elucidate the processes related to heart regeneration and to devise strategies that may be of potential value for treatment of human cardiac diseases. Since changes in energy metabolism are the central elements in the regenerative capacity (Eming et al., 2017; Nakada et al., 2017), and instead of focusing on single entities, we adapted a systems biology approach to generate in-depth knowledge of the transcriptomic, proteomic and metabolomic changes that occur at the tissue level in the extensively studied neonatal mouse heart model. The metabolomic profile of a tissue is a good representation of the final response to both autocrine and paracrine signaling, and combining proteomics and metabolomics to quantify changes in metabolites and their corresponding enzymes offers a complementary read-out for data interpretation that can advance our understanding of the mechanisms of regeneration (Mayr et al., 2007). In this work, we have demonstrated the differential regulation of 263 metabolites as well as mapped 366 proteins and 2,175 genes whose expression is differentially regulated during neonatal heart development. We linked these changes to metabolic reprogramming in the neonatal mouse heart during the first week of life. Moreover, we characterized in detail several cellular pathways and processes underlying neonatal heart development and regeneration, and pinpointed the fructolytic pathway to be predominant and functionally linked to increased cardiomyocyte proliferation in 1-day-old neonatal mice.

## Materials and methods

### Animals

All animal experiments were made according to the European Community guidelines for use of experimental animals and approved by the Finnish National Animal Experiment Board (permission number ESAVI/6718/04.10.03/2012). Hearts of postnatal day 1 (P1) and day 7 (P7) Hsd:ICR(CD-1®) male mice were harvested. The atrial appendages were removed, hearts were flash frozen in liquid nitrogen and stored until processing at −80°C.

### Sex determination of neonatal mice using qRT-PCR

A qRT-PCR assay that exhibits specificity for the sex-determining region of the Y chromosome was designed using a set of primers specific to the murine *Sry* gene (GenBank NM_011564.1 sequence). Isolation of genomic gDNA from mouse livers of the same animals was done with a Genomic DNA from tissue kit (Macherey-Nagel, Düren, Germany). Quantitative real-time reverse transcription (RT)-PCR was performed using LightCycler® 480 SYBR Green I Master Mix (Roche) with 50 ng of template gDNA according to manufacturer's instructions. gDNA from an adult male and female mouse served as controls.

### Whole transcriptomic analysis by RNA-seq

The hearts were snap frozen in liquid nitrogen and processed with the Precellys® 24 homogenizer with ceramic beads (Bertin Technologies). RNA from four P1 and four P7 mouse hearts was isolated with TRIzol as described (Rio et al., 2010), followed by DNAse I treatment. Agilent Bioanalyzer RNA pico chip (Agilent) was used to evaluate the integrity of RNA and Qubit RNA-kit (Life Technologies) to quantitate RNA. From each sample, a 1.5 μg of total RNA was collected, ribodepleted and further prepared to RNA-seq library by using ScriptSeq v2™ Complete kit (Illumina, Inc., San Diego, CA, USA). RNA-seq libraries were purified with SPRI beads (Agencourt AMPure XP, Beckman Coulter, Brea, CA, USA). The library QC was evaluated on High Sensitivity chips by Agilent Bioanalyzer (Agilent). Paired-end sequencing of RNA-seq libraries was done on an Illumina HiSeq platform (HiSeq 2000, Illumina, Inc., San Diego, CA, USA) using 100 cycles in each direction. The bioinformatic analysis of RNA-seq data was carried out by Exiqon A/S (Vedbaek, Denmark; currently Qiagen, http://www.exiqon.com) and included alignment of reads to the mouse reference genome (GRCm38; annotation reference: Ensembl_70), feature summation, and normalization of expression estimates. To allow the comparison of expression estimates between samples, the normalized expression values for each transcript were calculated as Fragments per Kilobase per Million Mapped Reads (FPKM). The expression profile of P7 heart samples were compared to the profile of P1 hearts; for each transcript the ratio between the average FPKMs was calculated and log2 transformed to log2FC-values. Transcripts showing a |FC| ≥1.5 and a False Discovery Rate (FDR) <0.05 were classified as differentially expressed. The list of all identified transcripts, indicating the differentially expressed genes (DEG) is provided in Tables S6C,D. The data were submitted to NCBI Gene Expression Omnibus (GEO) and are available under the accession number GSE107760 (release date 01.12.2018).

### Validation of fructolytic pathway with quantitative PCR

For quantitative PCR analyses, the RNA was isolated with TRIzol as described above, and reverse-transcribed using Bioline SensiFAST cDNA synthesis kit according to the manufacturer's instructions. Primer sequences of qPCR primers were as follows: *Slc2a1*(*Glut1*), 5′-CAGTTCGGCTATAACACTGGTG-3′ and 5′-GCCCCCGACAGAGAAGAT-3′, *Khk-c*, 5′-GCGTGGATGTGTCTCAGGTG-3′ and 5′-TGTTGACGATGCAGCAAGA-3′; *Slc16a3* (*Mct4*), 5′-GCCACCTCAACGCCTGCTA-3′ and 5′-TGTCGGGTACACCCATATCCTTA-3′; *Pfkfb2*, 5′-CGGGAATGGATCTACACTGG-3′ and 5′-GGAGAGCAAAGTGAGGGATG-3′, 18S 5′-ACATCCAAGGAAGGCAGCAG-3′ and 5′-TTTTCGTCACTACCTCCCCG-3′. Quantitative real-time PCR reactions were prepared with Roche FastStart Essential DNA Green Master as recommended by the manufacturer and run on a LightCycler 96 instrument (Roche). C_t_-values were normalized to the housekeeping gene 18S. Relative gene expression values were calculated as described (Livak and Schmittgen, 2001).

### Proteomic analyses

#### Sample preparation and proteolytic digestion

Individual frozen hearts of 1 day old male mice (*n* = 6) and 7 day old male mice (*n* = 6) were homogenized for 25 s at 4°C in 7 M urea, 2 M thiourea, 4% CHAPS using a Precellys® 24 homogenizer (Bertin Technologies). Samples were centrifuged for 1 min at 16.000 g. Ten micrograms of total protein lysate amounts were taken to be processed for digestion, using modified FASP protocol as previously described (Scifo et al., 2015).

#### Quantitative liquid chromatography tandem mass spectrometry HDMS^E^

For HDMS^E^ analysis, 300 ng of digested proteins/replicate (3 replicate runs per sample) was injected into the column and processed as described in Laakkonen et al. (2017).

#### Database mining, quantification and statistical analysis

Relative quantification between samples using precursor ion intensities was performed with Progenesis QI for Proteomics™ Informatics for Proteomics software (Non-linear Dynamics/Waters) and ProteinLynx Global Server (PLGS v3.0) (Laakkonen et al., 2017). Database searches were carried out against *Mus musculus* UniProtKB-database (release 2014_11, 33214 entries) with Ion Accounting algorithm using the following parameters: peptide and fragment tolerance: automatic, maximum protein mass: 500 kDa, min fragment ions matches per protein ≥7, min fragment ions matches per peptide ≥3, min peptide matches per protein ≥1, primary digest reagent: trypsin, missed cleavages allowed: 2, fixed modification: carbamidomethylation C, variable modifications: deamidation of asparagine and glutamine (NQ) residues, oxidation of methionine (M) and FDR < 4%. All of the identifications were subsequently refined to only *Mus musculus* identifiers, and protein lists were simplified by protein grouping. Protein quantitation was performed entirely on non-conflicting features, using precursor ion intensity data and standardized expression profiles. The stringency of accepted protein leads was increased by limiting the ratio between P7 as compared to P1 (differentially expressed proteins, DEP; fold change, |FC| > 1.5, computed from averaged, normalized protein intensities, and *p* < 0.05 by ANOVA for all comparisons, two unique peptides for quantitation).

### Protein bioinformatics analyses

The lists of up/down regulated protein changes with their corresponding, unique UniProtKB-database identifiers served as inputs into Ingenuity Pathways (IPA, Ingenuity systems, Redwood City, CA; www.ingenuity.com), with a main focus on Canonical pathways, and Disease and Function annotation. The Benjamini-Hochberg (B-H) multiple correction test was used to correct the *p*-values. Only most meaningful functional annotations, showing the lowest FDR were evaluated, furthermore, only z-scores ≥+2 (predicted activation) or ≤-2 (predicted inhibition) were considered (Pezzini et al., 2017). The functional annotation of terms was performed with ClueGO (v. 2.3.2; Bindea et al., 2009), utilizing as input DEP and KEGG/Reactome Pathway and Gene Ontology biological process annotation, and kappa score threshold of 0.4. The parameters relevant to the ClueGO analysis are reported in Table S2. The scatter and hierarchical clustering plots were drawn with Orange software (v.3.3; Demsar et al., 2013), while functional networks were drawn with Cytoscape (v.3.3; Shannon et al., 2003). The mitochondrial assignments were performed as described before (Mäkelä et al., 2016; Tikka et al., 2016), by utilizing mitochondrial annotations from IMPI database (http://www.mrc-mbu.cam.ac.uk/impi).

### Global metabolomic analysis

#### Sample preparation

P1 and P7 male mouse hearts were represented by four samples each, where each sample was a pool of four hearts (due to minimal tissue requirements of Metabolon, Inc.). Mouse hearts were shipped to Metabolon, Inc. (Durham, NC) and following receipt, samples were immediately stored at −80°C. Sample preparation was carried out as described previously at Metabolon, Inc. (Evans et al., 2009).

#### Non-targeted global metabolomic mass spectrometry analysis

Non-targeted global metabolomic MS analysis was performed at Metabolon, Inc. Extracts were subjected to a UPLC-MS/MS platform, which utilized a Waters Acquity UPLC with Waters UPLC BEH C18-2.1 × 100 mm, 1.7 μm column and Q-Exactive high resolution/accurate mass spectrometer (Thermo Scientific), interfaced with a heated electrospray ionization (HESI-II) source and Orbitrap mass analyser operated at 35,000 mass resolution, and processed as described in Evans et al. (2014).

#### Compound identification, quantification, data curation and statistical analysis

Metabolites were identified by automated comparison of the ion features in the experimental samples to a reference library of chemical standard entries that included retention time, molecular weight (*m/z*), preferred adducts, and in-source fragments as well as associated MS spectra and curated by visual inspection for quality control using software developed at Metabolon as described (DeHaven et al., 2010). Welch's two-sample *t*-test was used to identify molecules that differed significantly between experimental groups (*p* ≤ 0.05).

### Transmission electron microscopy

Cardiac samples (~1 mm^2^) for transmission electron microscopy were harvested from the left ventricular wall, fixed in 2,5% EM grade glutaraldehyde in 100 mM sodium-phosphate buffer for 2 h followed by a 24 h stabilization in fresh 100 mM sodium-phosphate buffer (4°C). Then, samples were post-fixed with 1% osmium tetroxide (1 h, RT), dehydrated with graded series of ethanol followed by acetone treatment and embedding in resin. Thin-sectioned samples were viewed and photographed with a Jeol JEM-1400 transmission electron microscope (Jeol Ltd., Tokyo, Japan). Mitochondrial number, volume fraction and trans-sectional area were analyzed with ImageJ software (v1.47, National Institute of Health, MD; Schneider et al., 2012). For the number of cristae per μm, the ImageJ line tool was used to draw a line across the stack of cristae, perpendicular to their orientation. The number of cristae crossing the line was counted and expressed in relation to the line length (Puente et al., 2014).

### High-resolution respirometry with oxygraph-2k

The mitochondrial respiration in the tissue was analyzed using the Oxygraph-2k (OROBOROS Instruments Corp., Innsbruck, Austria). Male mice hearts were excised as described above, and samples were prepared according to the manufacturer's Oxygraph-2k respirometer instructions. Briefly, 5 mg of tissue was homogenized in a SG3 shredder (Pressure Biosciences Inc., MA) at −20°C for 10 s (setting 1) and 5 s (setting 2) in 500 μl ice-cold MiRO_6_ buffer (0.5 mM EGTA, 3 mM MgCl_2_·6H_2_O, 60 mM K-lactobionate, 20 mM taurine, 10 mM KH_2_PO_4_, 20 mM HEPES, 110 mM sucrose, 1 g/l BSA, 280 u/ml catalase, pH 7.1). The contents were then further diluted with 4.5 ml MiRO_6_ buffer, of which 2.5 ml was transferred to each of the two chambers of an Oxygraph-2k instrument and allowed to incubate for 20 min before starting the substrate-uncoupler-inhibitor titration (SUIT) protocol (Pesta and Gnaiger, 2012).

### Immunoblotting, immunocyto- and immunohistochemistry

Mice hearts from six biological replicates of male P1 and P7 mice were harvested as described above and homogenized for 20 s at 4°C in RIPA Lysis and Extraction Buffer (Thermo Scientific) supplemented with protease inhibitors (Complete mini, Roche, Mannheim, Germany) using a Precellys® 24 homogenizer (Bertin Technologies) with ceramic beads. Protein content from vortexed and centrifuged samples was determined with Pierce BCA protein assay kit (Thermo Fisher Scientific, Monza, Italy). For Western blotting, 15–25 μg of protein lysates were resolved on Bolt™ 4–12% Bis-Tris Plus gels (Invitrogen, Carlsbad, CA) using SDS-PAGE under denaturing conditions and then transferred onto nitrocellulose membranes in an iBlot® Dry Blotting System (Invitrogen, Carlsberg, CA). The membranes were incubated 1 h at RT or O/N in blocking solution (5% bovine serum albumin or non-fat dry milk in TBS/0.1%Tween20). Incubation with primary antibodies was performed overnight (4°C): β-actin- Abcam 8227, GSK-3β- Cell Signaling Technology 9315, Rac1- Abcam 33186, Cdc42-Abcam 64533 and RhoA- Abcam 54835, followed by incubation with HRP conjugated antibodies (BioRad). Chemiluminescent detection was performed with Pierce ECL2 Western blotting substrate (ThermoFisher Scientific, Rockford, IL) according to manufacturer's instructions. Data were assessed using ImageJ software (Schneider et al., 2012) and each band normalized to its own loading control (Gapdh- Abcam 9485). Data were reported as mean percentages ± s.e.m. from all repeats, where the average of 1 day old mice was set to 100% and the data for P7 hearts expressed as percentages in relation to P1.

Proliferative cardiomyocytes were identified from paraffin-embedded sections of P1 (*n* = 10) and P7 (*n* = 7) mice hearts. Deparaffinized sections were stained with a Ki-67 antibody (SP6) (1:100, ThermoFisher Scientific, Fremont, CA) and anti-myosin heavy chain antibody (1:200, Millipore 05-716) for visualization of proliferative cells and cardiomyocytes, respectively. The number of Ki-67 and/or MHC positive and the total number of cells were counted from medium magnification (x400) immunofluorescent microscopy images with ImageJ software (Schneider et al., 2012). Statistical analyses were performed using Mann-Whitney *U*-test, considering significant *p*-values ≤ 0.05 as ^*^, *p*-values ≤ 0.01 as ^**^ and *p*-values ≤ 0.001 as ^***^.

### Hypoxia experiments

Neonatal mouse cardiomyocytes were isolated at P1. Briefly, hearts were collected into ice-cold PBS and isolated with neonatal heart dissociation and isolation kits following the manufacturer's instructions (Miltenyi Biotec, Germany). Experiments were performed with maintenance medium supplemented with 15 mM glucose, 25 μM fructose, 10% FBS and 5 units of penicillin and 5 μg of streptomycin per mL. Cells were exposed to hypoxia (3% of O_2_, OxyCycler, BioSpherix, NY) for 24 h. Control cells were cultured in regular 5% CO_2_ cell incubator under normal condition. For immunostaining, the cells were fixed with 10% paraformaldehyde and Ki-67 antibody (clone SP6) (1:100, ThermoFisher Scientific, Fremont, CA) and anti-myosin heavy chain antibody (1:200, Millipore 05-716) were used for visualization of proliferative cells and cardiomyocytes, respectively. The number of Ki-67 and/or MHC positive and the total number of cells were counted from medium magnification (400x) immunofluorescent microscopy images with ImageJ software (Schneider et al., 2012). Statistical analyses were performed using Mann-Whitney *U*-test, considering significant *p*-values ≤ 0.05 as ^*^, *p*-values ≤ 0.01 as ^**^ and *p*-values ≤ 0.001 as ^***^.

## Results

### Proteomic profiling of the neonatal mouse heart

**Figure 1:**
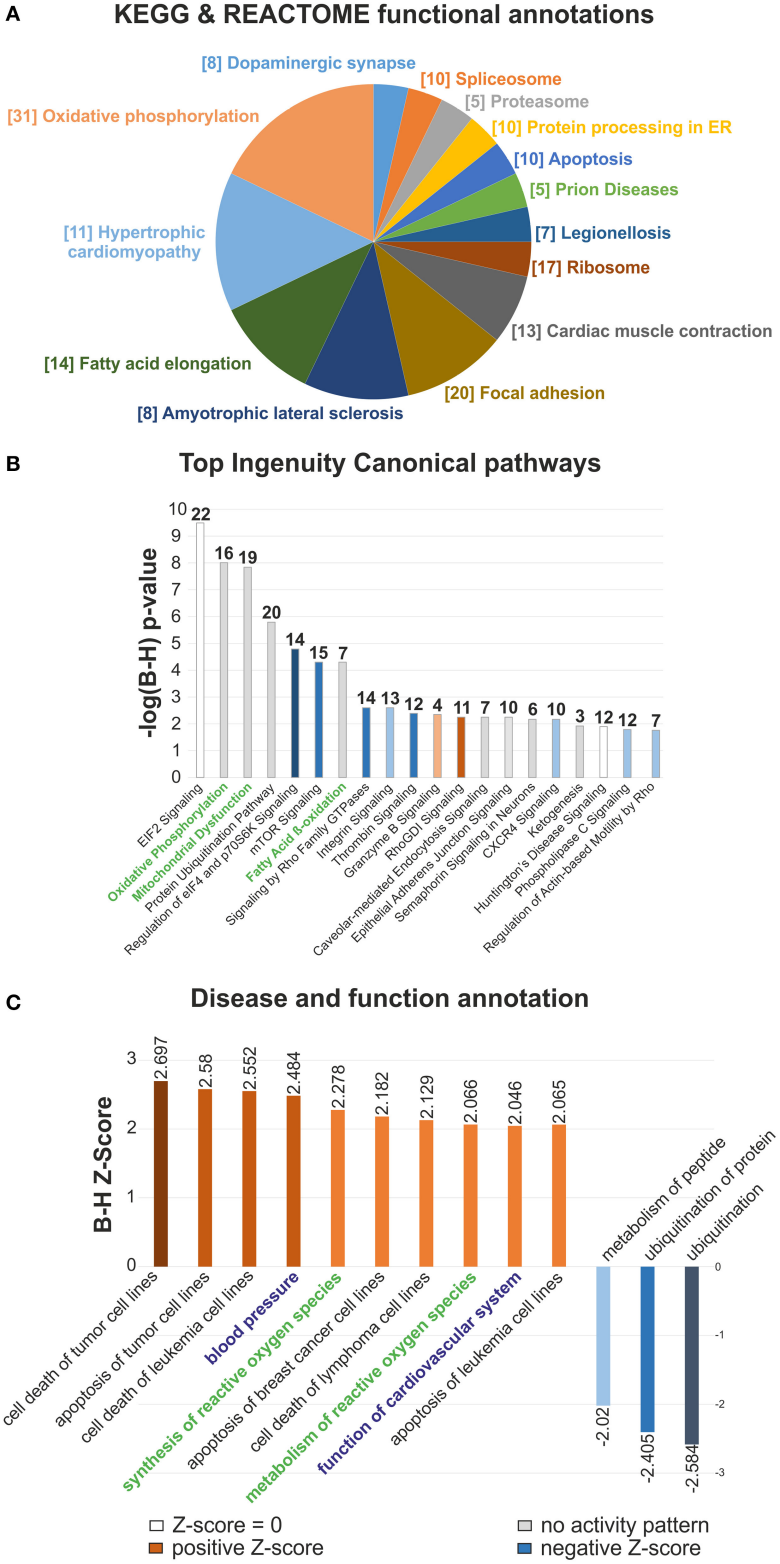
Clustering and functional analyses of differential proteomic data in the developing mouse heart. **(A)** Term enrichment analysis of the differentially expressed proteins by KEGG and Reactome Pathway. The size of pie chart sections proportionally represents the number of terms assigned to each group, with the most significant term per group indicated. The number of DEP assigned to each functional group is reported in brackets, **(B)** Top Ingenuity Canonical pathways chart of DEP with B-H adjusted *p*-values, **(C)** Disease and function IPA bar chart. The statistically enriched associations were sorted according to significant *z*-score values (|*z*| > 2). Functions in **(C)** related to Oxidative phosphorylation, Mitochondrial dysfunction and *Fatty acid* β*-oxidation* I are indicated in green. Function of Cardiovascular system, Blood pressure and Synthesis and Metabolism of reactive oxygen species categories are highlighted in blue and green, respectively. *n* = 6.

In order to elucidate the molecular basis of the regenerative capacity of the neonatal heart, a label free global proteomic analysis was performed at first. A total of 1,937 proteins in the neonatal mouse heart was identified and quantified, of which 366 proteins were differentially expressed (>1.5-fold change, FC; *p* < 0.05 and ≥2 unique peptides) between 1 (P1) and 7 (P7) day old mice. A complete survey of the identified proteins is provided in the Table S1, in which DEP were marked. In subsequent steps we functionally annotated the DEP using Kyoto Encyclopedia of Genes and Genomes (KEGG) and Reactome databases (Figure 1A and Table S2), revealing several processes related to *Cardiac muscle contraction, Cardiac hypertrophy, Oxidative phosphorylation, Fatty acid elongation, Focal adhesion* and mRNA/protein expression/degradation changes that were statistically enriched and associated with differential expression changes at P7 stage. The more detailed picture of functional enrichments is provided in Figure S1 (KEGG/Reactome pathway associations, bar chart view), Figure S2 (Gene Ontology Biological process, GO BP) and Table S2. Utilizing Ingenuity canonical pathways (IPA) classification, we subsequently revealed that *Oxidative phosphorylation, Mitochondrial dysfunction* and *Fatty-acid ß-oxidation I* were highly upregulated at P7 in comparison to P1 hearts, as indicated by their logP~-8 *p*-values (Figure 1B and Table S3). Moreover, *mTOR signaling, eIF4 signaling* and *Thrombin signaling* were found to be statistically significantly downregulated in P7 hearts. Disease and functions IPA annotation (Figure 1C) showed significant upregulation of multiple pathways associated with cell apoptosis, including *Cell death of tumor cell lines* (70 DEP associated)*, Apoptosis of tumor cell lines* (56 DEP), and *Cell death of leukemia cell lines* (18 DEP), all with B-H corrected *z-*scores >2.5 (Table S4). Other functions predicted to be strongly associated included *Synthesis* and *metabolism of ROS* (24 and 27 associated DEP; see also Figure S3), *Blood pressure* (14 associated DEP, B-H *z*-score 2.484) and *Function of cardiovascular system* (23 associated DEP, B-H *z*-score 2.046). In summary, novel pathways linked to cardiovascular function with putative connections to cell proliferation were identified, as well as a switch to an oxidative state with an increased ROS formation in the developing neonatal heart.

### Validation of differentially expressed protein targets associated with cardiovascular functions

**Figure 2:**
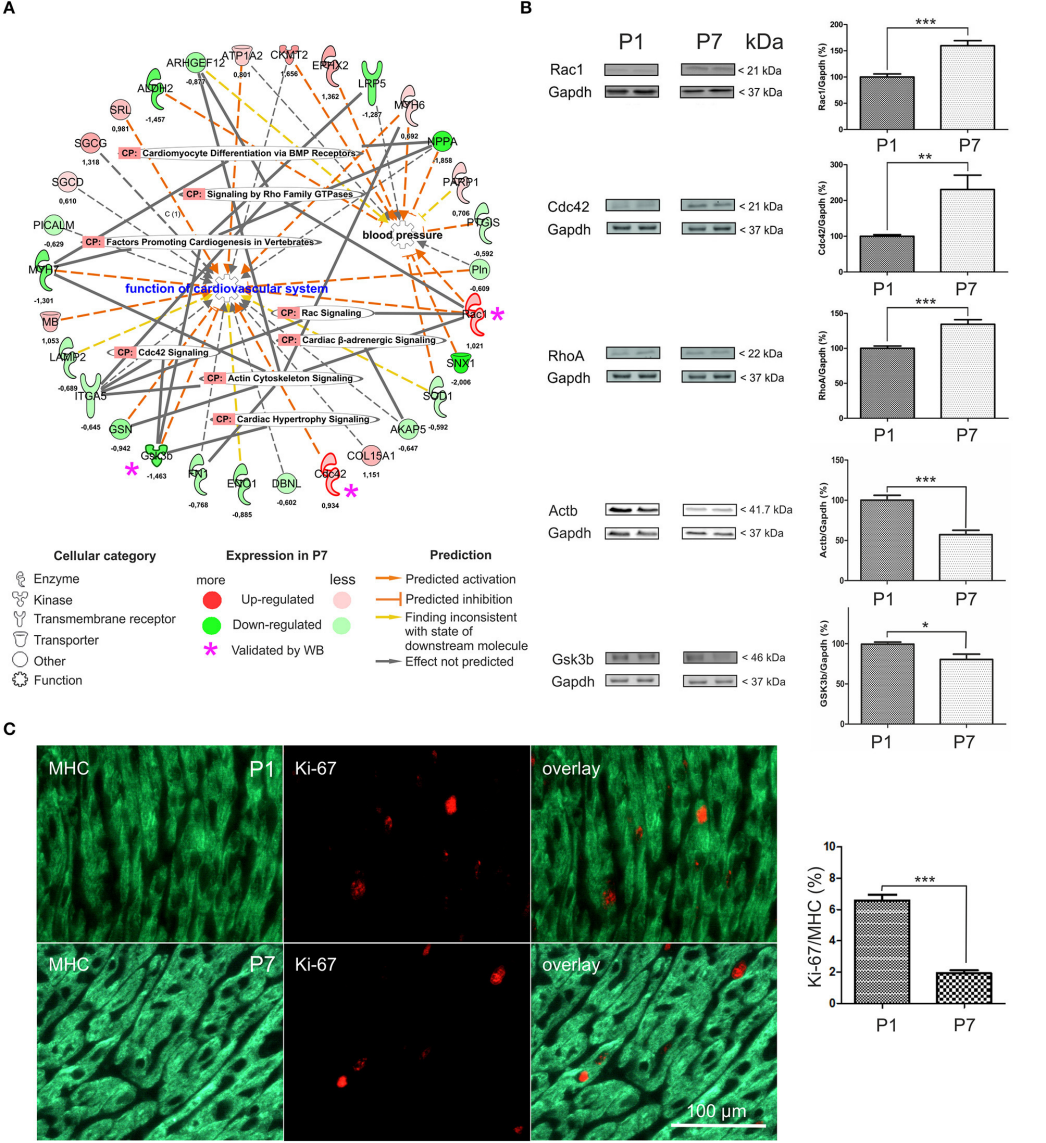
Functional annotation network analysis encompassing differentially expressed proteins in the mouse heart. **(A)** Eighteen down-regulated DEP and twelve up-regulated ones, including Rac1, Cdc42, and Gsk3b were functionally linked to *Function of cardiovascular system, Rac* and *RhoA family GTPase* and *Cdc42 signaling*, and *Cardiac hypertrophy signaling*. CP-canonical pathways. Log_2_FC-values of expression change are indicated, **(B)** Western Blot validation of differential expression data. P1- 1 day, P7- 7 days. *p < 0.05, **p < 0.01, ***p < 0.001 (heteroscedastic 2-tailed *t*-test), *n* = 6, **(C)** Representative Ki-67 and MHC stained samples of P1 and P7 myocardial tissues indicative of the amount of proliferative cardiomyocytes. *n* = 10 (P1) and *n* = 7 (P7), respectively. Mann-Whitney *U*-test, ***p < 0.001. 3262 (P1) and 2,747 (P7) cells / per group were counted.

To complement the functional bioinformatics analyses in the developing heart, we specifically focused on a network encompassing *Function of cardiovascular system* and *Blood Pressure*. A total of 30 DEP was associated with these functions, out of which 12 were significantly upregulated and 18 downregulated (Figure 2A and Table S5). These DEP were further linked to Canonical Pathways including *Cardiac hypertrophy, Actin cytoskeletal, Cdc42, Cardiac* β*-adrenergic*, and *Rac and Rho signaling pathways*, as well as *Cardiomyocyte differentiation via BMP receptors*. In order to validate the observed changes from mass spectrometry analysis, we confirmed the expression of some DEP associated with this network by semi-quantitative immunoblotting on total heart lysates. We selected two upregulated DEP (Rac1 and Cdc42) and one downregulated one (Gsk3b; Figure 2A) with connections to hypertrophy, as well as RhoA (a key player in RhoA signaling cascade, quantified with one unique peptide) and Actb (a key driver of actin cytoskeleton dynamics; Table S1). Immunoblotting with antibodies against Rac1 (Figure 2B) revealed bands at 21 kDa and 59.8% ± 9.4 increase in the signal intensity (*p* < 0.001) in P7 mice hearts. Antibodies against Cdc42 demonstrated a 130.6% ± 40.2 increase in the P7 mice hearts (*p* < 0.01). RhoA was detected at 22 kDa and band intensity analysis showed a 34.4% ± 6.5 increase in the P7 mice hearts. Immunoblotting with anti-Gsk3b antibodies revealed bands at 46 kDa and a band intensity decrease by 18.9% ± 7.1, in the P7 mice hearts. Actb was detected at 41.7 kDa, and band intensity analysis showed a 42.8% ± 8.3 decrease in the P7 mice hearts. In summary, the expression changes of selected proteins was verified and showed a similar direction with similar significant *p*-values. Immunohistochemical staining (Figure 2C) revealed a significant decrease in the expression of a proliferation marker, Ki-67 (*p* = 0.0008, Mann-Whitney *U*-test) in P7 cardiomyocytes as compared to P1, further supporting the switch from proliferation to growth by hypertrophy (Soonpaa et al., 1996; Leu et al., 2001; Porrello and Olson, 2014). Negative controls (primary antibody omitted) as well as staining of adult mice hearts showed no Ki-67 staining (data not shown). Taken together, we validated that cessation of proliferation in neonatal heart is accompanied by upregulation of RhoA/Rac1/Cdc42 cytoskeletal signaling related to cardiovascular function and hypertrophic growth.

### Results of RNA-seq profiling in the neonatal mouse heart validate the proteomic data

As another validation for the proteomics experiments, we performed the RNA-seq analyses of P1 and P7 stage whole hearts from an independent set of mice (Figures S4A,B). Altogether eight libraries were prepared from four P1 and P7 samples and subjected to sequencing on the Illumina platform. The paired-end sequencing of the libraries generated in total 401.349473 million 101-base-pair reads and allowed the identification of altogether 6.133 annotated and 46.231 unannotated transcripts (Tables S6A–D), of which 2.175 and 411 were deemed as statistically significantly differentially expressed (DEG), respectively. The correlation analysis of the expression and proteomic data in turn revealed 1,680 common entries present in both datasets (Table S6E). While a majority of these common entries were unchanged in their expression between P1 and P7 stages in both experiments, 35 DEP and DEG showed a similar and 17 a distinct expression change (marked in red and blue, respectively, Figures S5A,B). Similar percentages of DEP (15.57%) and (11.66%, DEG) were found to be unique for each assay type (Figure S5B). Further mining of the annotated DEG revealed a list of >350 cardiac genes that were strongly upregulated (*z-*score > 2) at the P7 stage. These genes were associated with functions like contractility, morphogenesis of endothelial tissue, and vasculogenesis (Table S7) and corroborated well the proteomic findings. For example, a very strong upregulation of *Signaling by Rho family GTPases* (*z-*score = 4.964) with 43 DEG was observed, providing a further support for the differential expression of this pathway that was also seen in the proteomic analysis (Table S7 and Figure S6). Similarly, *Production of Nitric Oxide and ROS* was strongly upregulated (with 35 DEG, *z-*score = 3.19), further validating the dysregulation of this pathway at the P7 stage (Table S7).

### Global metabolomic profiling of the neonatal mouse heart

**Figure 3:**
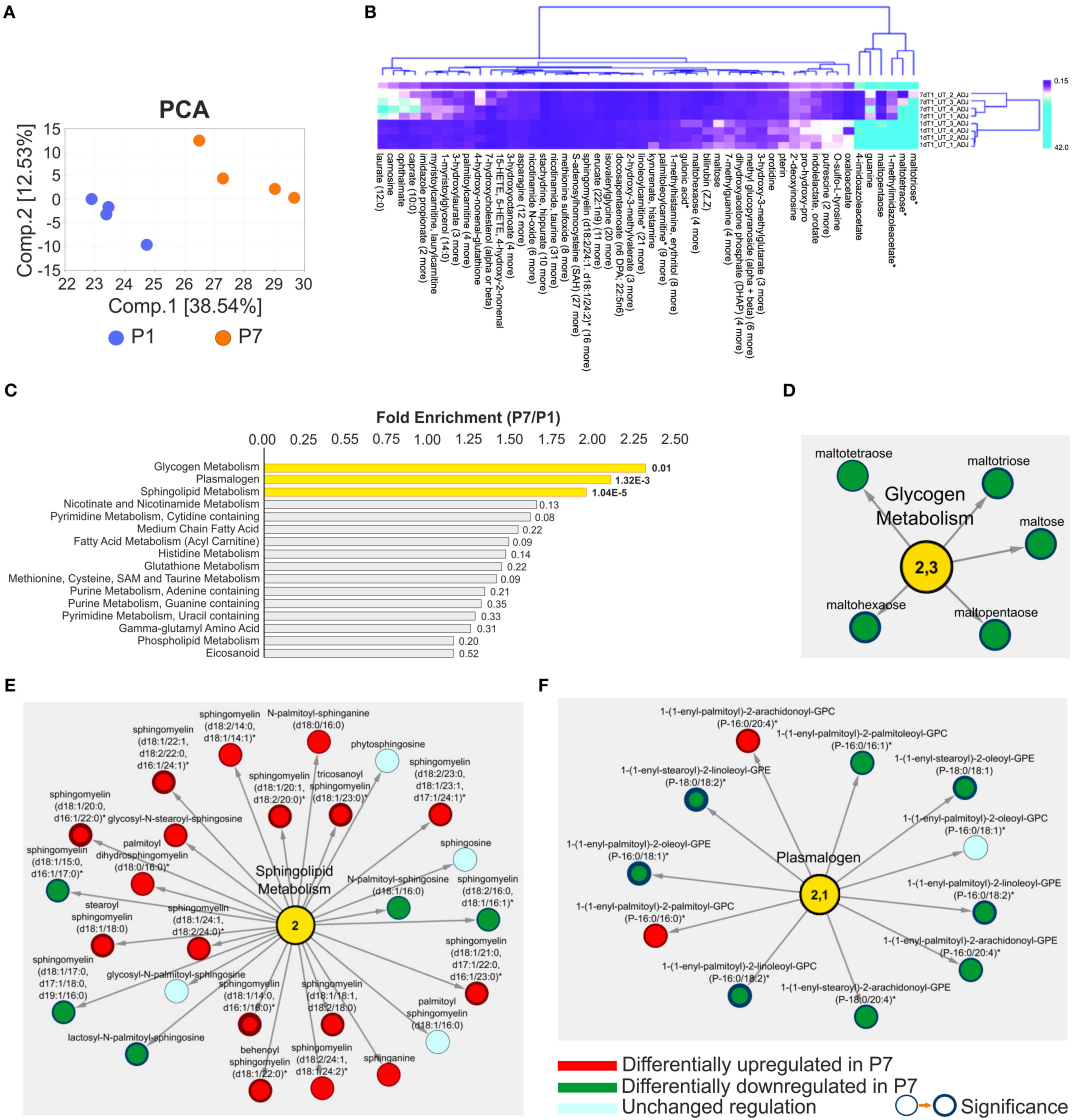
Presentation of untargeted global profiling analyses of metabolomic data in the developing heart (P7/P1). **(A)** Principal component analysis of metabolomic data demonstrating good separation and data grouping between measurement days. The percentage of variance is explained by the first and second principal components (Comp.1 and Comp.2), shown in brackets. **(B)** Hierarchical clustering of differential metabolomic data with compound and data grouping; asterisks denote metabolites with the highest fold change. Clustering by *k*-means including 50 clusters and leaf ordering, **(C)** Functional enrichment analysis of differential metabolomic data ranked by the Fold Enrichment (FE), in yellow statistically enriched categories are highlighted, FE ≥ 2 (P7/P1). Network presentations of the functional enrichments including **(D)** *Glycogen metabolism*, FE = 2.3, **(E)** *Sphingolipid metabolism*, FE = 2.0, and **(F)** *Plasmalogen*, FE = 2.1. The changes in metabolites abundance and directions are indicated. Node border thickness is proportional to significance (*p-*values). *n* = 16, including four pools of four.

A global metabolomic profiling of the mouse hearts was also used to enlighten the molecular basis of the loss of the regenerative capacity. Revealed in the analysis was in total 612 metabolites (Table S8). Among these, 263 metabolites with perturbed abundance (*p* ≤ 0.05) were identified, including 128 with increased and 135 with decreased abundance. Principal component analysis revealed a clear separation of the P1 and P7 mice metabolomic profiles (Figure 3A), a finding supported also by the unsupervised hierarchical clustering analysis (Figure S7). To gain insight into which biological processes were most affected by the heart development, a heat map of the global metabolomic profiling data set was sorted by metabolite classification (differentially regulated metabolites, DRM) among P1 and P7 stages. This map illustrates both the direction and significance of change of metabolites among the various pair-wise comparisons across the P1 and P7 hearts. It can be noted that the glycogen metabolites maltotriose, maltotetraose and maltopentaose, as well as the histamine metabolites 1-methylimidazoleacetate and 4-imidazoleacetate were highly abundant in P1 hearts (Figure 3B). The functional linkage analysis of DEMs to KEGG pathways identified *Glycogen metabolism* as well as *Sphingolipid* and *Plasmalogen metabolism* as statistically enriched pathways (FE ≥ 2, E range10^−5^−10^−2^, Figure 3C). The network depiction of these pathways with corresponding DRM is illustrated in detail in Figures 3D,F. The *Glycogen metabolism* and *Plasmalogen* functional modules were significantly decreased (5 and 11 metabolites, including 8 DRM with significantly lowered abundance, respectively), while *Sphingolipid metabolism* was significantly enriched in P7 hearts (17 upregulated DRM encompassing sphingomyelin metabolism). Plasmalogens and other phospholipids (Table S8) were more abundant in P1 mice, pointing to an increased cell membrane biosynthesis, which is required for proliferation. These results also strengthen the view that P1 mice are relying on glycolytic metabolism with increased glycogen storage.

### Key enzymes and metabolites involved in mitochondrial oxidative respiration are elevated in the 7 day mouse heart

**Figure 4:**
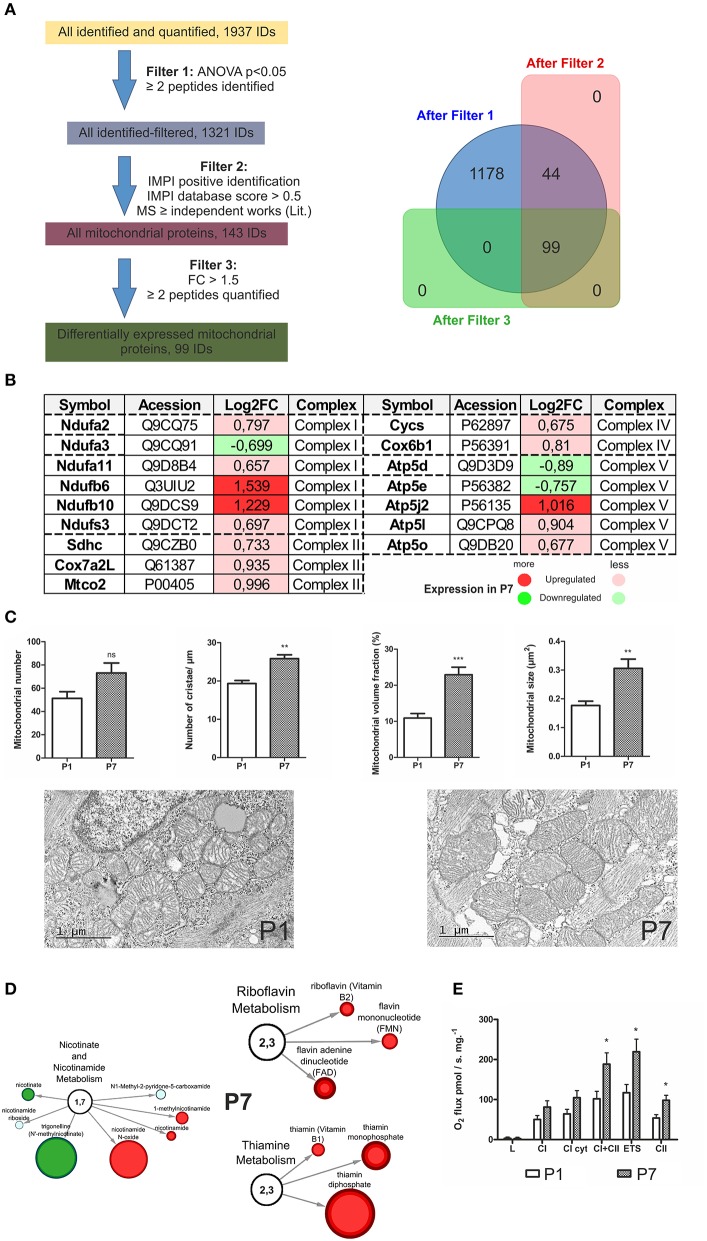
Mitochondrial sorting of proteomic and metabolomic data in the developing heart. **(A)** Scheme of mitochondrial assignment of proteomic data with bioinformatics filtering steps; IMPI—*the mitochondrial proteome database*, **(B)** Portrayal of mitochondrial chain complexes (I–V) with assigned, differentially expressed proteins, **(C)** Electron microscopy depiction of heart mitochondrial morphology at P1 and P7 stage. Although the number of mitochondria is relatively unchanged (P7 vs. P1), the size, mitochondrial volume and the number of cristae increase over time. **p < 0.01, ***p < 0.001 by Mann-Whitney-test, **(D)** Network presentations of the mitochondrial metabolites with underlying processes indicated. The changes in metabolites abundance are indicated. In red-upregulation, in green-downregulation. Size of the node corresponds to an increasing significance (*p*-values), **(E)** Oxygen flux measurements with Oroboros. L, resting state; CI cyt, complex I after addition of cytochrome C; CI+II, after activation of complex II with succinate; ETS, electron transfer system, Complex II (after inhibition of complex I by rotenone). *n* = 6, *p < 0.05, Mann-Whitney *U*-test.

Since the *Mitochondrial dysfunction, Oxidative phosphorylation* and *Synthesis of ROS* functional categories were statistically enriched in P7 hearts, we further mined the databases (see section Materials and Methods and Mäkelä et al., 2016; Tikka et al., 2016) to assign all mitochondrial proteins and distinguish DEP of interest. Using this strategy, we assigned 99 mitochondria-associated DEP (see scheme in Figure 4A and Table S1B). It can be noted that several members of mitochondrial chain complexes were altered in P7 hearts (Figure 4B), with members of complex I, Ndufb6 (log_2_FC = 1.539) and Ndufb10 (log_2_FC = 1.229) showing the highest upregulation levels, respectively. As the number and structure of mitochondria in a cell is related to its activity, we subsequently examined the morphological differences in heart mitochondria at the P1 and P7 stage (Figure 4C). The mitochondrial volume fraction and mitochondrial size were markedly larger in the cardiomyocytes of P7 mice as compared to the P1 ones. The number of mitochondrial cristae, indicating the mitochondrial ability for energy production, was also significantly higher in the P7 hearts. In accordance with their roles in the electron transport chain, metabolomic studies showed an increase in riboflavin metabolism (Figure 4D), with a significant increase (values expressed as P7/P1) in both *riboflavin* and *flavin mononucleotide* as well as *flavin adenine dinucleotide. Nicotinamide, nicotinamide N-oxide* and *1-methylnicotinamide* in the nicotinamide metabolism pathway were also elevated. Furthermore, *thiamine*, which serves as a co-factor for Krebs' cycle enzymes, was found to be strongly upregulated together with the levels of *thiamine mono-* and *diphosphate*. Finally, respirometric measurements showed a consistent increase in oxygen flux through the electron transport chain in P7 hearts (Figure 4E). These results further support the switch to oxidative phosphorylation at the P7 stage.

### Comparative proteomic and metabolomic analysis of elevated fatty acid β-oxidation in the 7 day mouse heart

**Figure 5:**
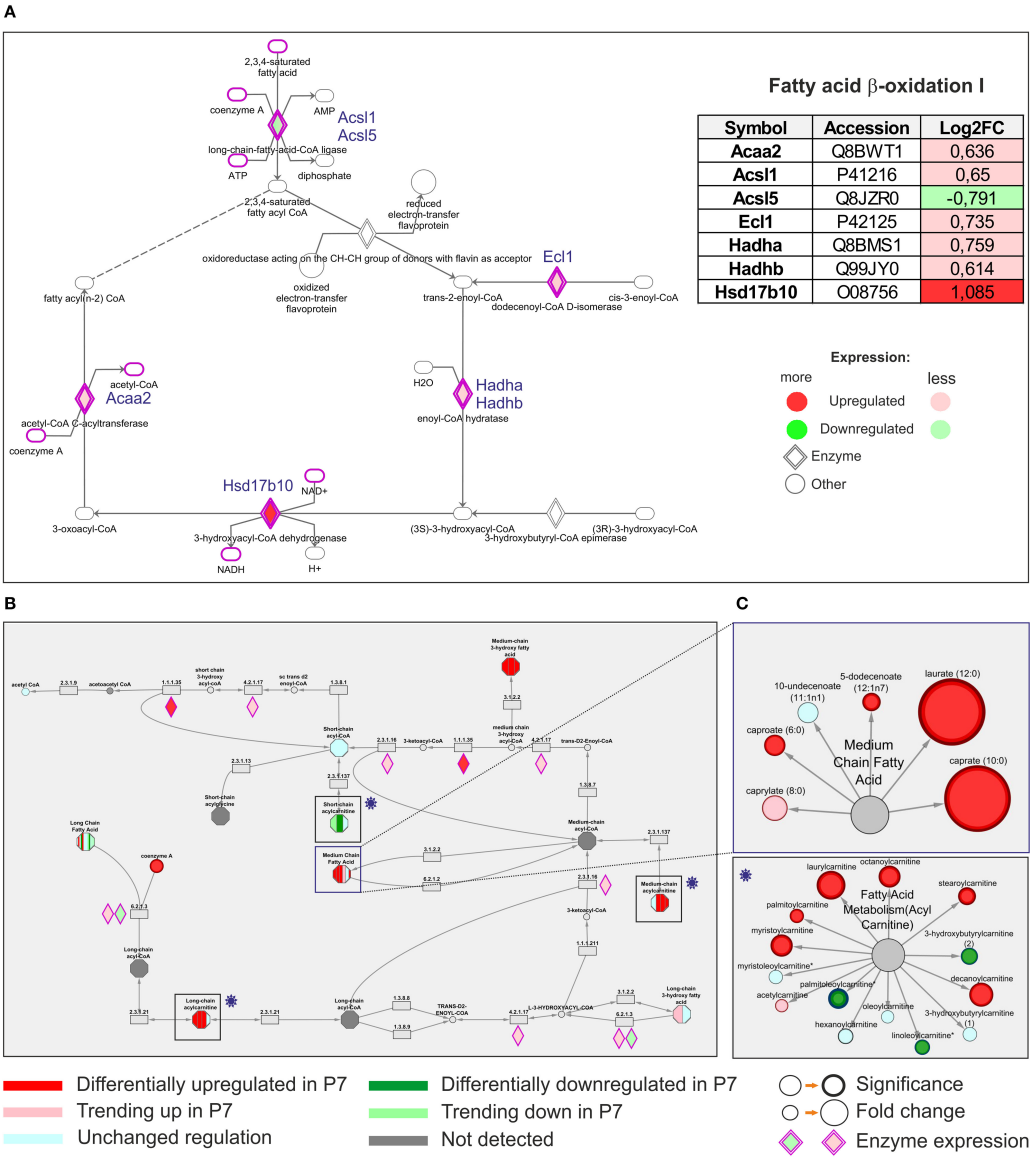
Comparative analysis of differentially expressed proteomic and differentially regulated metabolomic data. **(A)** Differentially expressed upregulated enzymes assigned to Fatty acid-β-oxidation pathway at 7 days (KEGG annotation), as indicated, **(B)** The same pathway was pinpointed with altered differential regulations of various metabolites, encompassing specifically *Fatty acid metabolism (Acyl carnitine)* and *Medium Chain fatty acid functional modules*. **(C)** The differentially regulated metabolites are indicated in subnetworks shown in rectangles on the right.

Under aerobic conditions the predominant substrates used by the adult mammalian heart are free fatty acids, with long-chain fatty acids being the major component. These fatty acids are coupled to carnitines and transported into the mitochondrial matrix by carnitine-O-palmitoyl-transferase (CPT) I. The acylcarnitines are reconverted to fatty acyl-CoA by CPT II, located in the inner membrane. Proteomic analysis revealed that both CPT I and II were upregulated in P7 heart (Table S1A). Following entry into the mitochondrion, fatty acids are converted to units of acetyl-CoA through the process of fatty acid β-oxidation (FAO). Querying the proteomic results against KEGG pathways demonstrated an association of a total of 23 proteins to the fatty acid degradation and among these the enzymes Acaa2, Acsl1, Ecl1, Hadha, Hadhb, and Hsd17b10, linked to FAO, were significantly upregulated in P7 mice hearts. Differentially expressed enzymes assigned to *Fatty acid* β*-oxidation I* pathway at P7 (KEGG annotation) are visualized in Figure 5A. Consequently, the P7 heart was characterized by an increased amount of metabolites involved in fatty acid synthesis (Table S8), transport (medium and long-chain *Acyl carnitines*) and FAO, such as *Medium chain fatty acids* (Figures 5B,C). Selected, DRM are indicated in subnetworks in Figure 5C, where sizes of nodes denote the level of statistical significance. Consistent with an increase in oxidative phosphorylation, the P7 hearts are utilizing fatty acids as their energy substrate.

### Proliferation is affected in hypoxia conditions

**Figure 6:**
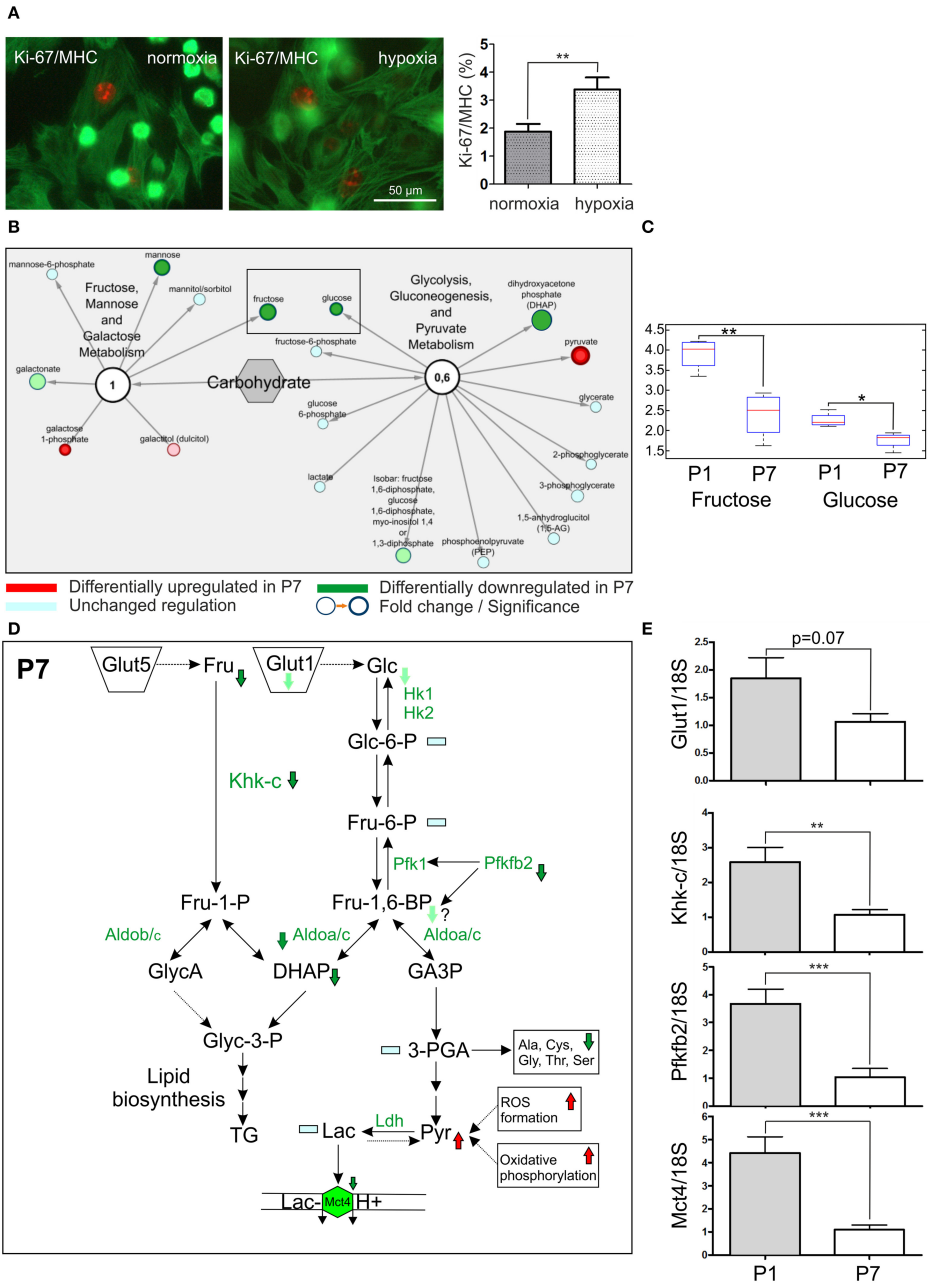
Proliferation of cardiomyocytes and fructolytic pathway are strongly affected in P7 hearts. **(A)** Hypoxia condition (3% O_2_) increases the cardiomyocyte proliferation rate. The cardiomyocytes were isolated from P1 hearts and kept in hypoxia chamber for 24 h. The number of proliferative cardiomyocytes stained by Ki-67 and MHC was counted. *n* = 10, respectively. Mann-Whitney *U*-test, *p < 0.05. 3407 (normoxia) and 3,072 (hypoxia) cells / per group were counted, **(B)** The network overview of sugar metabolism with two functional modules depicting *Fructose, mannose and galactose metabolism* and *Glycolysis, gluconeogenesis and Pyruvate metabolism*. The downregulation of fructose in P7 hearts was observed in metabolomics experiments, marked by rectangular, **(C)** The representation of a fructose downregulation in P7 hearts. Box and whiskers plot, the median, lower 25% and upper 75% percentiles and minimum and maximum values are shown. Welch's Two-Sample *t*-test, **p < 0.01, *p < 0.05. Glucose levels are shown in comparison, **(D)** The overview of the fructolysis/glycolysis pathways with affected targets. The downregulated metabolites (Fructose, Fru; dihydroxyaceton phosphate, DHAP), enzymes (Phosphofructokinase-2, Pfkfb2*;* ketohexokinase isoform c, Khk-c*;* Aldolase a/c, Aldoa/c measured in MS^E^ experiments) and transporters (Glut1/Slc2a1; Mct4/Slc16a3) are indicated with an arrow. Lac, lactate; Pyruvate (Pyr), Mct4, monocarboxylate transporter, LDH, lactate dehydrogenase, **(E)** The downregulation of *Glut1, Mct4, Khk-c* and *Pfkfb2* at the mRNA level was assessed by q-PCR, by comparing the RNA abundance from tissue samples derived from P1 and P7 hearts. C_t_ values were normalized to the level of a housekeeping gene 18S in each measurement, *n* = 10. Mann-Whitney *U*-test, ***p < 0.001.

Previous studies have demonstrated that inhibition of aerobic respiration by systemic hypoxemia alleviates oxidative DNA damage and reactivates the endogenous regenerative properties of the adult mammalian heart (Nakada et al., 2017). We utilized the hypoxic chamber to mimic fetal physiological hypoxic conditions, and harvested the P1 hearts, from which the cardiomyocytes were isolated. Exposure to low oxygen (3% O_2_) for 24 h resulted in an increased proliferation of cardiomyocytes as judged by immunodetection with Ki-67 and MHC (3.4% vs. 1.9%, *p* < 0.05; Figure 6A). These findings support the notion that targeting hypoxia signaling may represent a novel therapeutic strategy for cardiac regeneration.

### Fructose metabolism and fructose-induced glycolysis are enhanced in the P1 hearts

Previous study by Park et al. (2017) provided compelling evidence that the unique anoxia resistance of the naked mole-rats is strongly dependent on metabolic rewiring under oxygen deprivation, and in particular, induction of fructose-induced glycolysis. Data from metabolomics experiments revealed marked upregulation of fructose metabolism in P1 hearts (Figures 6B,C). Therefore, we reasoned that metabolic rewiring toward fructose-induced glycolysis could explain, at least in part, enhanced cardiomyocyte proliferation and endogenous regenerative capacity of the neonatal mice (Figure 6D). In P1 hearts, the mRNA expression of 6-phosphofructo-2-kinase/fructose-2.6-bisphosphatase 2 (Pfkfb2), an activator of phosphofructokinase 1 (Pfk1) and a rate-limiting enzyme of cardiomyocyte glycolysis, was 3- to 4-fold increased as compared to P7 hearts (Figure 6E). Furthermore, the mRNA expression of the key fructose-metabolizing enzyme ketohexokinase-C (Khk-c), the isomer displaying superior affinity for fructose as compared to Khk-a, was also increased by 2- to 3-fold in P1 hearts (Figure 6E). Finally, the mRNA expression of hypoxia-inducible lactate transporter Mct4, responsible for proton symport-mediated lactate efflux (Jones and Morris, 2016), was increased more than 4-fold in P1 hearts (Figure 6E). In line with these findings, transcriptomic analyses revealed a strong association of HIF1α signaling to a number of DEG (24 DEG, Table S7). HIF1α activation of SF3B1-dependent splicing of KHK enforces fructolysis to promote cardiac hypertrophy in response to pathologic stress (Mirtschink et al., 2015). Taken together, these data reveal enhanced fructose-driven metabolism and fructose-induced glycolysis as the key metabolic pathways in the neonatal mouse heart. These differences may also help explain the intriguing endogenous regenerative capacity of the neonatal mice heart.

### Changes in the amino acid metabolism underlie the transition and differences in energy utilization

**Figure 7:**
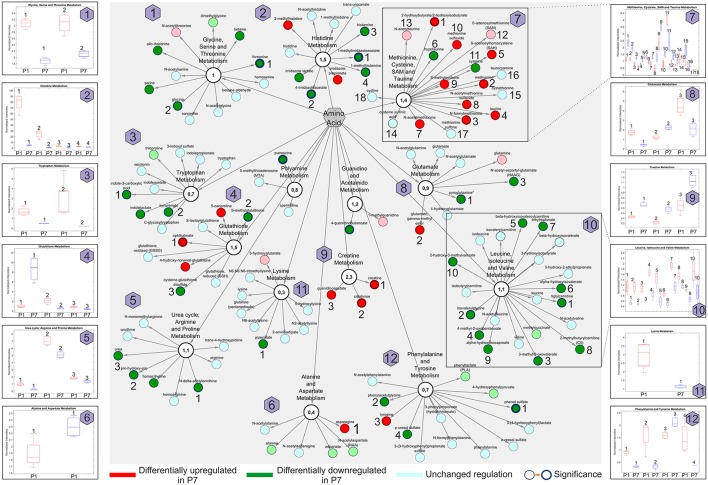
Amino acid network presentations of global metabolomic analysis of the developing heart **(P7/P1)**. Pathways with differentially regulated metabolites are indicated. Insets indicate the normalized intensities of individual amino acids (numbered) in selected metabolic pathways; P1 (red) and P7 (blue). *n* = 16, including four pools of four hearts. Node border thickness is proportional to significance (*p-*values).

In subsequent steps, we searched for specific regulators of the regenerative capacity and turned our attention to amino acids whose metabolism in the neonatal heart has not been studied previously. Changes in amino acids between P1 and P7 stages are plotted in Figure 7 and listed in detail in the Table S8.

Overall, amino acids allocated to the Methionine, cysteine, SAM and taurine metabolism, Creatine and Glutathione metabolism were significantly enriched in P7 hearts, whereas amino acids belonging to the Histidine metabolism, Leucine, isoleucine and valine metabolism, the Glycine, serine and threonine metabolism and the Urea cycle encompassed many members with prominently lowered abundance. Selected metabolites from sub-networks and the normalized intensities are shown as insets. In summary, we pinpointed 12 various functional amino acid network modules comprising various DRM with altered abundance.

## Discussion

Current treatments for patients suffering a myocardial infarction are only palliative and the loss of massive amounts of cardiomyocytes in the injured heart, accompanied by fibrotic scar formation, increases the risk of heart failure and cardiovascular mortality. The development of novel strategies to enhance endogenous tissue regeneration and repair after MI is thus required. The neonatal mouse heart at P1 has an exceptional, inherent capacity to regenerate after a myocardial infarction, a capacity lost by 7 days of age (P7). The neonatal heart represents an important model to elucidate the mechanisms behind the heart's regenerative switch (Porrello et al., 2011; Haubner et al., 2012). Better understanding of physiological development in the postnatal period of the mouse heart, specifically the shift from hyperplasia to hypertrophy, as well as of the metabolic changes in energy demand, may contribute to successful future elucidation on how to activate regenerative molecular mechanisms in adult mammalian hearts. Moreover, revealing such mechanisms may provide targets for local stimulation or reactivation of cardiomyocyte proliferation in adult tissue (Foglia and Poss, 2016). Since the combined use of proteomics together with metabolomics and transcriptomics permits a holistic view to a biological system, we sought to explore the postnatal development using these techniques to understand the intrinsic repair mechanism. Moreover, to our knowledge, this is the first study combining in-depth proteomic, metabolomic and transcriptomic analyses in the neonatal heart model.

Several in-depth proteomic investigations have been targeted at generating detailed inventories of diverse mouse tissues, including heart, using a multitude of different analysis strategies (reviewed in Kislinger and Gramolini, 2010). Decreased proteolytic capacity and accruing proteotoxicity have been suggested to directly exacerbate outcomes in heart failure, cardiac infarcts and hypertrophy (Wang and Robbins, 2006; Li et al., 2011). We have compared the results of relative protein abundance analyses at P1 and P7 stage with the results of a large dataset (performed with the usage multi-step subcellular purifications and various enrichment techniques) of protein turnover dynamics in the heart of six common genetic strains of adult mice (however not including the strain used in our study), acquired under both normal and hypertrophic conditions (Lau et al., 2016). The majority of our proteomic data was retained in a data set provided by Lau et al., with 1,817 common proteins (Figure S8). Similarly, we related our proteomics data to a peptidomics data set from neonatal mouse heart at the same stages (Fan et al., 2017). Three out of four cardiac-related precursor proteins, corresponding to differentially expressed peptides, whose expression was validated by the authors by quantitative PCR at the mRNA level displayed the same trends of change in their expression (Myh7, Hadha and Hspb1; Table S1), while one, Cox8b was not found in our dataset (one differentially expressed peptide found by Fan et al., 2017). The database searches linked 30 DEP to cardiovascular functions at P7 (Table S5 and Figures 2A,B). Among these were members of Rho/Rac/Cdc42 signaling cascades, molecular switches implicated in cytoskeletal dynamics, cell movement and polarity, differentiation, contraction and motility and other common cellular functions, important for spatiotemporal fine tuning of numerous cardiovascular functions and cardiovascular development (Loirand et al., 2013). Our data support the neonatal heart transition from hyperplastic to hypertrophic growth (Figure 2C), which is consistent with the reported correlation between the proliferative capacity of cardiomyocytes and their regenerative potential (Jopling et al., 2010; Haubner et al., 2012; Porrello et al., 2013; Senyo et al., 2013). Moreover, KEGG/Reactome functional association identified processes related to hypertrophy (Figure 1A) as statistically significant and associated with differential expression changes at P7. During the first week of the neonatal heart development, a significant loss of Ki-67 positive, proliferating cardiomyocytes was seen. We also annotated other signaling pathways that may be important regulators of the regenerative potential of the neonatal heart. IPA canonical pathway analysis predicted upregulated activity of *mTOR, eIF4* and *Thrombin signaling* in P1 mice, as well as increased *PI3K/AKT signaling* (Table S3), all of which are associated with increased cell proliferation (Ma and Blenis, 2009) and cell-cycle regulation (Figure 1C) (Laplante and Sabatini, 2009; Vivien et al., 2016). In addition, mTOR signaling is also a key regulator of cell metabolism (Laplante and Sabatini, 2009). Metabolomic amino acid analysis identified some additional, putative regulators of the proliferative response. High abundance of histidine, histamine and histamine metabolites was observed in P1. Histamine can serve as an autocrine growth factor in cancer, stimulating cell proliferation (Rivera et al., 2000). Furthermore, an increase in S-adenosylmethionine conversion to S-adenosylhomocysteine was observed (Figure 7). S-adenosylhomocysteine plays a significant role in regulating nucleotide and protein expression through methylation and is important for cell function and survival by altering cell-cycle kinetics to arrest cell growth (Boal et al., 2011). These pathways represent previously unreported, potentially interesting targets for local pharmacological activation of cardiomyocyte proliferation in heart regeneration.

During the neonatal period there is a shift in the energy utilization from proliferating cardiomyocytes depending on glycolysis in P1 hearts to cardiomyocytes relying on oxidative phosphorylation in P7 hearts, which similarly to the adult ones utilize fatty acids as its primary fuel source under most conditions (Lopaschuk and Jaswal, 2010). Taking a closer look at molecules involved in energy metabolism, we found that the majority of enzymes assigned to fatty acid β-oxidation (FAO) and Krebs cycle were upregulated in P7 hearts, whereas glycolytic enzymes were downregulated. Our findings corroborated the results by Puente et al. (2014) and showed that ~80% of enzymes detected in both studies display the same direction in their expression. Furthermore, our metabolomic profiling revealed that the P7 hearts exhibited a significant increase in the majority of medium chain fatty acids and acylcarnitines, the metabolites which are formed in order for long-chain fatty acids to be transported from cytosol to the mitochondrial matrix for FAO. These results are, therefore, consistent with significant changes in fatty acid transport and β-oxidation rates in P7 hearts, further reinforced by the upregulation of multiple enzymes involved in FAO, measured in HDMS^E^ proteomic experiments (Figure 5 and Table S1). Interestingly, in a recent study in which adult mice were subjected to normoxic or hypoxic conditions post MI, hypoxia induced metabolic reprogramming of adult cardiomyocytes, resulting in cell-cycle re-entry and myocardial regeneration (Nakada et al., 2017). Multiple-omics analyses of these hearts showed striking similarity to our data, with hypoxic animal and our neonatal P1-omics data having similar directionality in their trends (Nakada et al., 2017), indicative of feasibility in reversal of glycolytic energy utilization in adult hearts. We also demonstrated that the shift to oxidative phosphorylation is accompanied by morphological differences in mice heart mitochondria and an increased number of cristae in P7 mouse hearts (Figure 4C) similarly as presented in Puente et al. (2014). Moreover, while the number of mitochondria did not significantly increase from P1 to P7 stage, both mitochondrial size and volume fraction were markedly increased in P7 mice hearts. Interestingly, our metabolomic analyses demonstrated significant increases in nicotinamide (NAM) and riboflavin metabolism in P7 hearts (Figure 4D). NAM is a precursor of nicotinamide adenine dinucleotide, which together with FADH_2_ serve as electron carriers in oxidative phosphorylation and cofactors for many dehydrogenases. NAM *N*-oxide (increased in P7) is a natural metabolite of NAM with no assigned biological function. High doses of NAM *N*-oxide have been reported to affect the differentiation of leukemia cells (Iwata et al., 2003), and metabolites of NAM clearance were proposed as potential biomarkers of peroxisome proliferator-activated receptor-α (PPAR) activation, having a key role in fatty acid β-oxidation (Zhen et al., 2007). Thiamine metabolism was also significantly upregulated (Figure 4D). Thiamine is a co-factor of Krebs cycle enzymes, such as pyruvate dehydrogenase and α-ketoglutarate dehydrogenase, important for regulating the flux of metabolites through the cycle. In addition, some amino acids feeding this cycle, such as tyrosine, asparagine, methionine, and glutamine were found at significantly higher levels in P7. Altogether these findings, in combination with multiple mitochondrial enzymes of the electron transport chain being significantly upregulated (Figures 4A,B), bioinformatic associations to oxidative phosphorylation (Figures 1A–C) and oxygen flux increase in the P7 hearts (Figure 4E), corroborated the shift to oxidative phosphorylation in P7 mouse hearts. In contrast, several enzymes and metabolites in carbohydrate utilization such as glucose and glucose-derived metabolites were abundant in P1 hearts (Table S8). Specifically, P1 hearts contained higher levels of glycolytic intermediates and glucogenic amino acid metabolites (Figure 7 and Table S8). In line with this, proteomic measurements showed higher abundance (at P1) of the glycolytic enzymes pyruvate kinase (Pklr), phosphoglycerate kinase 2 (Pgk2) and α-enolase (Eno2) (Table S1A). Correspondingly, glycogen metabolism was significantly upregulated in P1 hearts. These results support the view similar to cancer cells; glycolytic energy utilization is a key driver behind the proliferative capacity. The metabolic reprogramming to oxidative phosphorylation and associated molecular mechanisms might, therefore, be critical for loss of the regenerative potential.

The naked mole-rat survives anoxia by substituting fructose for glucose as the energy substrate for anaerobic metabolism in the heart and brain (Park et al., 2017). Such a switch to fructose-driven glycolytic respiration avoids feedback inhibition of glycolysis via phosphofructokinase (Park et al., 2017). We report here that fructose metabolism and fructose-induced glycolysis are enhanced in neonatal mice heart under normoxic conditions. Our results also suggest that neonatal mice heart utilizes proton symport-mediated lactate efflux to avoid intracellular lactate accumulation and alterations in intracellular pH level. Previously, Mirtschink et al. have demonstrated that myocardial hypoxia activates fructose metabolism via HIF-1α-SF3B1-KHK-C axis in human and mice models of pathological cardiac hypertrophy (Mirtschink et al., 2015) underlying the importance of fructose metabolism in the regulation of cardiomyocyte growth in adults. Interestingly, MCT4 expression is induced by hypoxia (Mirtschink et al., 2015) and upregulated in many proliferating cancer cells (Jones and Morris, 2016), whereas MCT4 inhibition has been shown to prevent hypoxic response, cell proliferation and tumor progression (Voss et al., 2017). When isolated cardiac myocytes from P1 neonatal mice were cultured under normoxia or under mimicked physiological hypoxia, a clear upsurge in cell proliferation in hypoxic condition was evident (Figure 6A). These results suggest the presence of a functional link between the observed fructose-induced glycolysis active at P1 and cardiomyocyte proliferation, an observation which warrants further in-depth exploration. Taken together, our results underline the key importance for cell metabolism in regulation of cardiomyocyte proliferation and innate cardiac regenerative capacity.

Although the majority of adult heart cardiomyocytes are terminally differentiated, cardiomyocytes may be capable, to a limited degree, of self-renewal in order to repair the daily micro-damages that occur in the adult heart. It has been shown that human cardiomyocytes have an approximate turnover rate of 1% per year, which declines further after the age of 50 (Bergmann et al., 2015). In neonatal mice, proliferation of existing cardiomyocytes is the primary mechanism behind the regenerative potential (Jopling et al., 2010; Haubner et al., 2012; Porrello et al., 2013). Although this native capacity is insufficient to compensate for large-scale tissue damage associated with MI, it is important to clarify the mechanisms that could trigger adult cardiomyocytes to de-differentiate, re-enter the cell-cycle and proliferate, in order to replace damaged cardiomyocytes after a MI (Porrello and Olson, 2014). An increase in the synthesis of ROS accompanies the shift to oxidative phosphorylation in the oxygen-rich postnatal environment, and postnatal increase in ROS formation induces cell-cycle arrest through activation of the DNA damage response (Puente et al., 2014). Likewise, the transcriptomic analysis revealed a massive change in the mitotic machinery in the postnatal heart, correlating very closely with shutting down of the cell-cycle (Haubner et al., 2012). Scavenging of ROS in cardiomyocytes or inhibiting the DNA damage response pathway delayed postnatal cell-cycle arrest of cardiomyocytes (Puente et al., 2014). Adult mice which suffered a MI and were exposed to hypoxia, showed significantly decreased ROS formation and improved heart regeneration (Nakada et al., 2017). ROS, therefore, have been proposed as important mediators in the shift from proliferating growth to terminally differentiated cells, which grow by hypertrophy in the postnatal mammalian heart (Puente et al., 2014; Nakada et al., 2017). Alike, we also demonstrated that multiple elements related to oxidative stress were upregulated in P7 hearts. IPA functional annotations recognized significant upregulation of *Synthesis* and *Metabolism of reactive oxygen* species (Figure 1C, Figure S3 and Table S7). Moreover, metabolomic profiling, identified novel regulators of oxidative stress, previously not linked to this setup. Increase of oxidative stress markers such as opthalmate, several gamma-glutamyl amino acids, 5-oxoproline, 2-hydroxybutyrate, and methionine sulfoxide (Figure 7), as well as a robust rise in lipid peroxidation mediators such as 4-hydroxy-2-nonenal (HNE) and 13-HODE and 9-HODE (Table S8) were observed in P7 hearts. Similarly, eicosanoids were highly abundant in P7. Label free HDMS^E^ quantitation revealed significant downregulation of the mitochondrial isoform of aldehyde dehydrogenase (ALDH2) in P7 hearts, which plays an important role in neutralizing HNE produced by ROS-induced lipid peroxidation (Table S1). Increased HNE accumulation can lead to myocardial dysfunction and heart failure, and modify essential cardiac survival signaling molecules (Mali and Palaniyandi, 2014). On the other hand, molecules with antioxidant properties such as N-acetylmethionine and taurine were elevated at P7 (Figure 7), likely in a cellular attempt to neutralize the generated ROS. This implies that the P7 hearts are characterized by an increase of oxidative stress likely counteracted by physiological antioxidants. Metabolomic analyses in P1 also revealed significant upregulation of plasmalogen metabolism (Figures 3E,F). Plasmalogens together with other phospholipids are major constituents of the cell membrane, and high abundance of phospholipids in P1 mice points to an increased cell membrane formation, consistent with an increased propensity for cell proliferation. Plasmalogens may have a protective role in P1 mice hearts since they have been shown to protect against ROS-induced lipid peroxidation. Furthermore, low levels of plasmalogens are associated with increased cardiovascular mortality (Zoeller et al., 1988; Stenvinkel et al., 2004), and sphingolipid metabolism is upregulated in P7 hearts under oxidative stress (Nikolova-Karakashian and Reid, 2011; de Faria Poloni et al., 2014). An upsurge in ceramide levels can activate nicotinamide adenine dinucleotide phosphate (NADPH) oxidase, which in turn aggregates to lipid rafts and together with Src and Rac1 forms functional redox signaling platforms. This, in consequence, activates ROS, increases hypertrophy, cytoskeletal remodeling and inflammation (Nikolova-Karakashian and Reid, 2011; de Faria Poloni et al., 2014). Increased NADPH oxidase activity has been revealed in P7 hearts, while its' activity was markedly decreased in adult mouse hearts subjected to hypoxia (Puente et al., 2014; Nakada et al., 2017). Furthermore, increases in NADPH oxidase activity can be functionally coupled to the Rho family GTP-ases: RhoA, Rac1 and Cdc42 (upregulated in P7, as measured in both transcriptomic and proteomics analyses; Figure 2 and Figure S6), which actively regulate actin cytoskeletal changes, thereby creating a putative link between oxidative stress and hypertrophic growth in the neonatal heart (Brown et al., 2006; Satoh et al., 2006).

**Figure 8:**
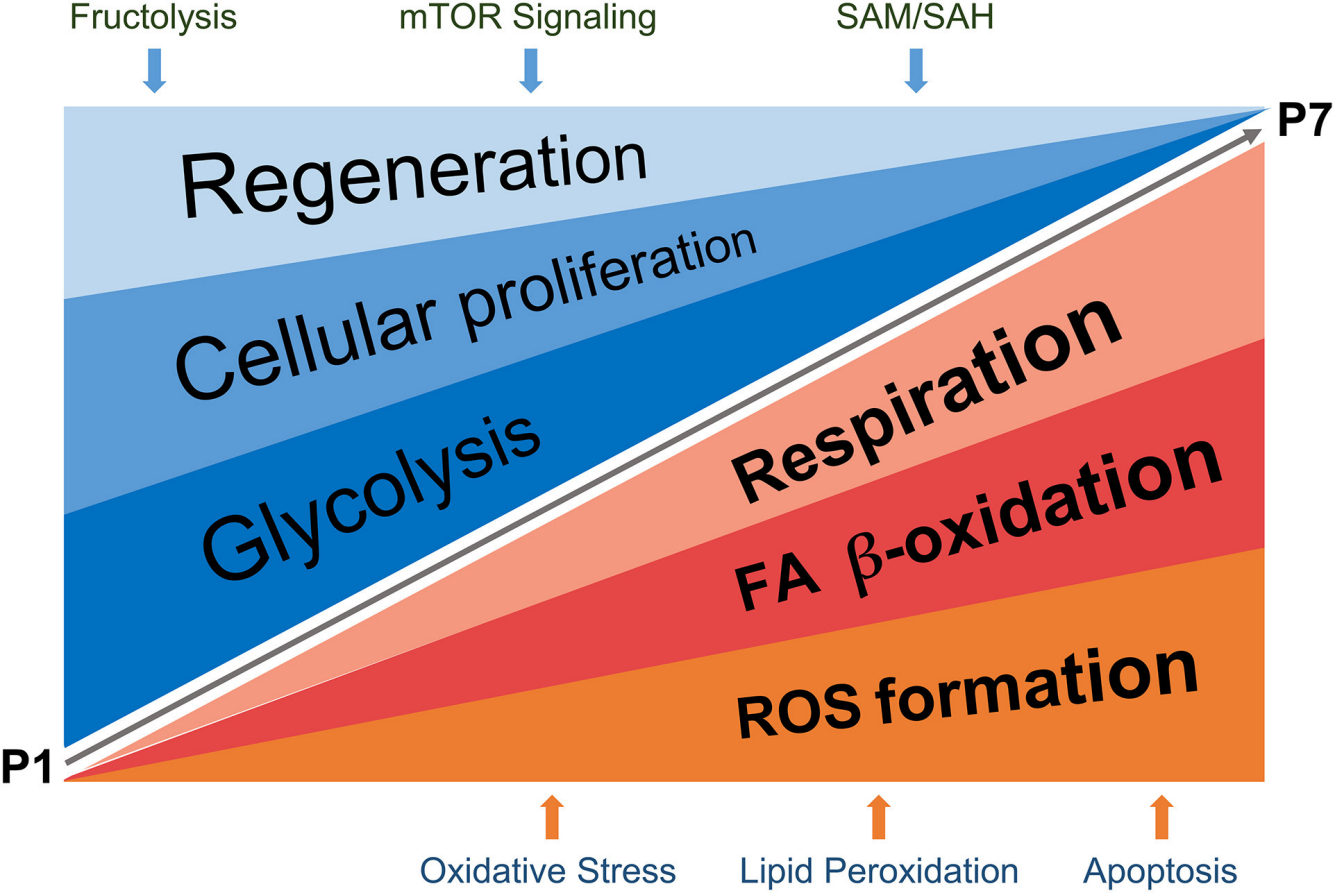
Schematic representation of proposed regulatory events detected in the study, taking place during P1–P7 neonatal heart development. The processes described in detail (in Results and Discussion sections) are summarized in the cartoon. SAM, S-adenosylmethionine; SAH, S-adenosylhomocysteine.

Altogether, using systems biology approaches we have mapped fundamental differences in energy utilization of the neonatal mouse heart during the first week of life and demonstrated that a transition from fructose-induced glycolysis under hypoxic conditions to oxidative phosphorylation, with a concomitant increase in physiological oxidative stress points to a switch from hyperplastic to hypertrophic growth. Furthermore, we pinpointed fructolysis, mTOR, plasmalogen, methionine and histidine metabolism, lipid peroxidation and sphingolipid signaling as novel pathways involved in these processes, suitable for pharmacological interventions (cartoon in Figure 8). We believe that the in-depth characterization of molecular processes taking place during the early postnatal days, a time period shown in strong correlation with literature to be specifically associated with loss of cardiac regenerative potential upon injury, will facilitate more targeted research on heart regeneration and paves way toward generation of new hypotheses when designing cardiac therapies.

## Author contributions

ML, SB, RS, MB, PL, EK, and EM: designed the research; SB, RS, DB, ST, MK, and PF: collected the data; ML, SB, RS, PF, GC, MK, and MT: analyzed the data; ML and SB: wrote the manuscript. All authors reviewed the manuscript.

### Conflict of interest statement

The authors declare that the research was conducted in the absence of any commercial or financial relationships that could be construed as a potential conflict of interest.
