# Supplementary material for: Characterizing the Key Metabolic Pathways of the Neonatal Mouse Heart Using a Quantitative Combinatorial Omics Approach
Source: Front Physiol. 2018 Apr 11;9:365. doi: 10.3389/fphys.2018.00365 (PMC5904546; doi:10.3389/fphys.2018.00365)
Supplement: Supplementary file 1 [file Image1.PDF]

Figure S1. Gene ontology Biological Processes Analysis. Gene Ontology annotation of Biological Process (*GO_BiologicalProcess-GOA_06.11.2016*) for the significant DEP (|FC|>1.5, p<0.05, ≥2 unique peptides) by ClueGO. Filtering at 0.01 p-value. Bar length represents the percentage of associated genes (proteins) for each GO term, while bar label indicates the number of identified DEP annotated to each GO term. Bars are differentially colored according to their association to functional GO group (shown in the pie chart).

Figure S2. KEGG and Reactome Pathway ClueGO representation of functional enrichments. Pathway and functional annotation of KEGG and Reactome Pathway *(KEGG_08.11.2016)* for the significant DEP (|FC|>1.5, p<0.05, ≥2 unique peptides) by ClueGO. Filtering at 0.01 p-value. Bar length represents the percentage of associated genes (proteins) for each term; bar label indicates the number of identified DEP annotated to each functional term. Bars are differentially colored according to their association with respective functional group (see pie chart in Figure 1B).

Figure S3. Functional annotation network analysis encompassing DEP with their association to ROS events.

Fourteen down-regulated and thirteen up-regulated DEP linked to *Synthesis* and *Metabolism of ROS*, and associated to the canonical pathways *Mitochondrial dysfunction* and *Production of nitric oxide and ROS in macrophages*. CP- canonical pathways.

Figure S4. PCA and Spearman correlation plots of transcriptomic profiles in P1 and P7 hearts. (**A**) Principal component analysis (PCA) plot for P1 and P7. Principal component analysis (PCA) of RNA-seq expression data. The PCA was performed using top 500 genes with the largest coefficient of variation and identified as expressed at each sample, PC- Principal component, (**B**) Heat map and unsupervised hierarchical clustering by sample and genes on the listed samples using the 500 genes that have the largest coefficient of variation based on FPKM counts. Hierarchical clustering was performed using Euclidean distance measure and average linkage; n=8.

Figure S5. Differential proteomics/transcriptomics data correlation plot. (**A**) Scatter plot for P1 and P7 demonstrating the log2 FC change P7/P1 ratio for differential proteomics and transcriptomics data. DEG- differentially expressed genes, DEP- differentially expressed proteins. The 35 entries with a similar trend of differential expression are indicated in red. Distinct trend of expression is

represented by 17 common entries shown in blue, (**B**) The Venn diagrams portraying the number and percentage of unique and common DEG and DEP, respectively. The corresponding data can be found in Table S6.

Figure S6. Network representation of *Signaling by Rho family GTPases* predicted to be upregulated by RNA-seq analysis. A strong upregulation of *Signaling by Rho family GTPases* (*z*-score= 4.964, by IPA® prediction) with predicted activation state and encompassing 43 differentially expressed genes in P7 hearts is depicted. The corresponding data can be found in Table S7.

Figure S7. Spearman correlation plot of metabolomic profile data in P1 and P7 hearts. Hierarchical clustering was performed using Euclidean distance measure and average linkage. n=16, including 4 pools of 4.

Figure S8. Comparative analysis of proteins identified in heart samples. Venn diagram of heart-expressed proteins, identified and quantified in this study (*1D-7D-all* and *1D-7D-2-p*) in comparison with the heart multi-step profiling study by (Lau et al., 2016), encompassing 6 mouse strains and quantified at ≥4 data points (however excluding the mouse strain used this our study). The number of unique and shared proteins among these studies is indicated. *All*- all proteins identified. *2-p*- ≤2 unique peptides and p≤0.05 used in $HDMS^E$ quantitation.

Table S1. The list of all proteins detected in $HDMS^E$ proteomic experiments with their fold changes and annotation. Differentially expressed proteins and mitochondrial assignments are indicated, (**B**) MITO_DEP- Differentially expressed proteins with mitochondrial assignments.

Table S2. The list of *GO Biological Process* and *KEGG* combined with *Reactome Pathway* functional annotations of differentially expressed proteins. (**A**) p0.01_Biological Process Gene Ontology annotations at 0.01 *p*-value threshold, (**B**) p0.01_KEGG ReactomePathway- Reactome pathway database annotations at 0.01 *p*-value threshold, (**C**) BP ClueGO Log- ClueGO- Gene Ontology Biological Process ClueGO processing information log, (**D**) KRP ClueGO Log- KEGG and Reactome databases processing information log.

Table S3. The complete list of Ingenuity Canonical Pathways associations of differentially expressed proteins. Ingenuity® database annotation.

Table S4. The complete list of Ingenuity Diseases and Functions associations of differentially expressed proteins. Ingenuity® database annotation.

Table S5. The list of Cardiovascular System Development and Functions annotations of differentially expressed proteins. Ingenuity® database annotation.

Table S6. The list of all transcripts detected by RNA-seq analysis of whole hearts with differentially regulated ones is indicated. n=8. (**A**) Summary of the mapping results for each sample, (**B**) Number of genes identified in each sample with a fragment count estimation of at least 10 counts per gene, (**C**) Number of transcripts and annotated genes identified in each sample, (**D**) Number of identified differentially expressed gene loci, (**E**) Number of identified common entries in both transcriptomics and proteomics analyses.

Table S7. The list of all canonical pathways associated with differentially expressed genes in neonatal heart. Differentially expressed genes (molecules) and corresponding *p*-values and *z*-scores are indicated. B-H- Benjamini-Hochberg correction. Ingenuity® database Pathway annotation.

Table S8. The list of all metabolites detected with differentially regulated metabolites and pathways indicated. (**A**) OrigScale- values were normalized in terms of raw area counts, (**B**) ScaledImpData- each biochemical in OrigScale was rescaled to set the median equal to 1. Missing values were imputed with the minimum; (**C**) Pathway heat map- database annotation of metabolomics data to cellular pathways.

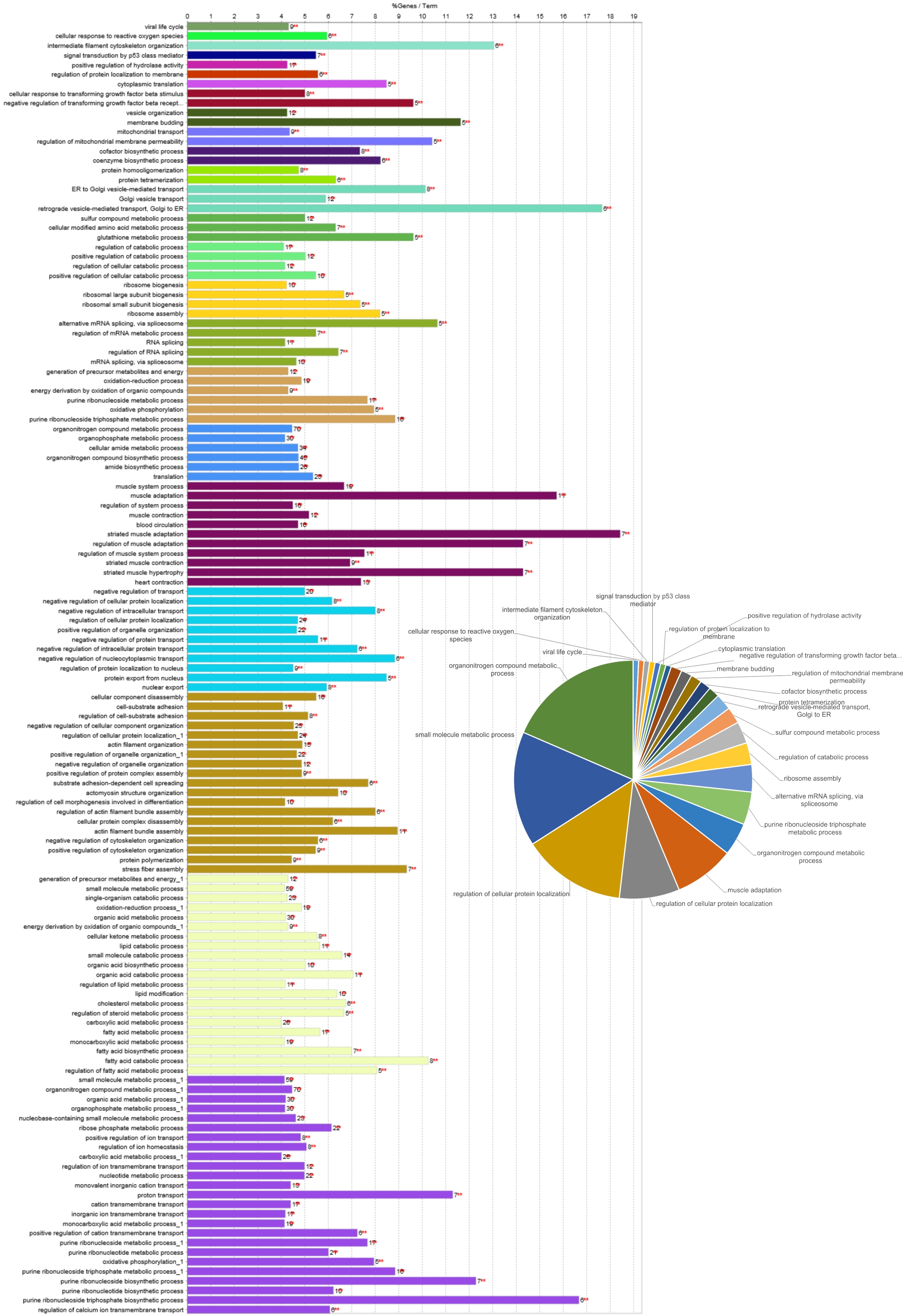
%Genes / Term
0 1 2 3 4 5 6 7 8 9 10 11 12 13 14 15 16 17 18 19
viral life cycle
cellular response to reactive oxygen species
intermediate filament cytoskeleton organization
signal transduction by p53 class mediator
positive regulation of hydrolase activity
regulation of protein localization to membrane
cytoplasmic translation
cellular response to transforming growth factor beta stimulus
negative regulation of transforming growth factor beta recept...
vesicle organization
membrane budding
mitochondrial transport
regulation of mitochondrial membrane permeability
cofactor biosynthetic process
coenzyme biosynthetic process
protein homooligomerization
protein tetramerization
ER to Golgi vesicle-mediated transport
Golgi vesicle transport
retrograde vesicle-mediated transport, Golgi to ER
sulfur compound metabolic process
cellular modified amino acid metabolic process
glutathione metabolic process
regulation of catabolic process
positive regulation of catabolic process
regulation of cellular catabolic process
positive regulation of cellular catabolic process
ribosome biogenesis
ribosomal large subunit biogenesis
ribosomal small subunit biogenesis
ribosome assembly
alternative mRNA splicing, via spliceosome
regulation of mRNA metabolic process
RNA splicing
regulation of RNA splicing
mRNA splicing, via spliceosome
generation of precursor metabolites and energy
oxidation-reduction process
energy derivation by oxidation of organic compounds
purine ribonucleoside metabolic process
oxidative phosphorylation
purine ribonucleoside triphosphate metabolic process
organonitrogen compound metabolic process
organophosphate metabolic process
cellular amide metabolic process
organonitrogen compound biosynthetic process
amide biosynthetic process
translation
muscle system process
muscle adaptation
regulation of system process
muscle contraction
blood circulation
striated muscle adaptation
regulation of muscle adaptation
regulation of muscle system process
striated muscle contraction
striated muscle hypertrophy
heart contraction
negative regulation of transport
negative regulation of cellular protein localization
negative regulation of intracellular transport
regulation of cellular protein localization
positive regulation of organelle organization
negative regulation of protein transport
negative regulation of intracellular protein transport
negative regulation of nucleocytoplasmic transport
regulation of protein localization to nucleus
protein export from nucleus
nuclear export
cellular component disassembly
cell-substrate adhesion
regulation of cell-substrate adhesion
negative regulation of cellular component organization
regulation of cellular protein localization_1
actin filament organization
positive regulation of organelle organization_1
negative regulation of organelle organization
positive regulation of protein complex assembly
substrate adhesion-dependent cell spreading
actomyosin structure organization
regulation of cell morphogenesis involved in differentiation
regulation of actin filament bundle assembly
cellular protein complex disassembly
actin filament bundle assembly
negative regulation of cytoskeleton organization
positive regulation of cytoskeleton organization
protein polymerization
stress fiber assembly
generation of precursor metabolites and energy_1
small molecule metabolic process
single-organism catabolic process
oxidation-reduction process_1
organic acid metabolic process
energy derivation by oxidation of organic compounds_1
cellular ketone metabolic process
lipid catabolic process
small molecule catabolic process
organic acid biosynthetic process
organic acid catabolic process
regulation of lipid metabolic process
lipid modification
cholesterol metabolic process
regulation of steroid metabolic process
carboxylic acid metabolic process
fatty acid metabolic process
monocarboxylic acid metabolic process
fatty acid biosynthetic process
fatty acid catabolic process
regulation of fatty acid metabolic process
small molecule metabolic process_1
organonitrogen compound metabolic process_1
organic acid metabolic process_1
organophosphate metabolic process_1
nucleobase-containing small molecule metabolic process
ribose phosphate metabolic process
positive regulation of ion transport
regulation of ion homeostasis
carboxylic acid metabolic process_1
regulation of ion transmembrane transport
nucleotide metabolic process
monovalent inorganic cation transport
proton transport
cation transmembrane transport
inorganic ion transmembrane transport
monocarboxylic acid metabolic process_1
positive regulation of cation transmembrane transport
purine ribonucleoside metabolic process_1
purine ribonucleotide metabolic process
oxidative phosphorylation_1
purine ribonucleoside triphosphate metabolic process_1
purine ribonucleoside biosynthetic process
purine ribonucleotide biosynthetic process
purine ribonucleoside triphosphate biosynthetic process
regulation of calcium ion transmembrane transport
signal transduction by p53 class mediator
intermediate filament cytoskeleton organization
positive regulation of hydrolase activity
regulation of protein localization to membrane
cellular response to reactive oxygen species
cytoplasmic translation
negative regulation of transforming growth factor beta...
viral life cycle
membrane budding
organonitrogen compound metabolic process
regulation of mitochondrial membrane permeability
cofactor biosynthetic process
protein tetramerization
retrograde vesicle-mediated transport, Golgi to ER
small molecule metabolic process
sulfur compound metabolic process
regulation of catabolic process
ribosome assembly
alternative mRNA splicing, via spliceosome
purine ribonucleoside triphosphate metabolic process
organonitrogen compound metabolic process
muscle adaptation
regulation of cellular protein localization
regulation of cellular protein localization

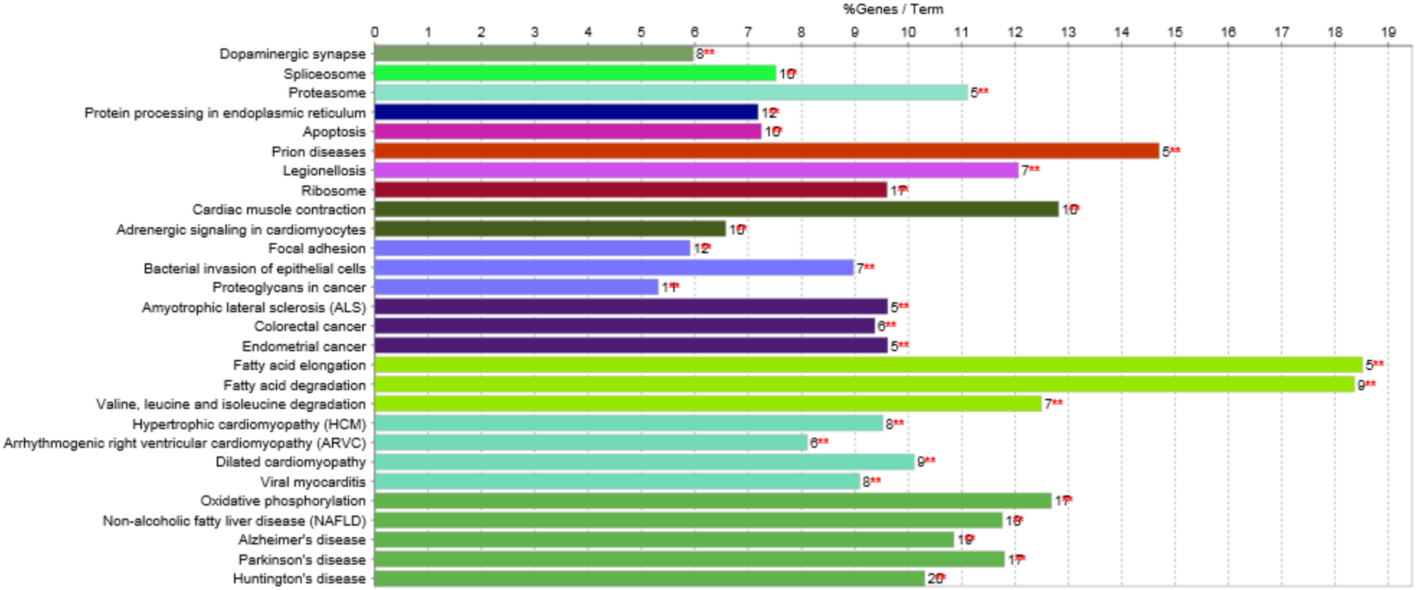
%Genes / Term
0
1
2
3
4
5
6
7
8
9
10
11
12
13
14
15
16
17
18
19
Dopaminergic synapse
Spliceosome
Proteasome
Protein processing in endoplasmic reticulum
Apoptosis
Prion diseases
Legionellosis
Ribosome
Cardiac muscle contraction
Adrenergic signaling in cardiomyocytes
Focal adhesion
Bacterial invasion of epithelial cells
Proteoglycans in cancer
Amyotrophic lateral sclerosis (ALS)
Colorectal cancer
Endometrial cancer
Fatty acid elongation
Fatty acid degradation
Valine, leucine and isoleucine degradation
Hypertrophic cardiomyopathy (HCM)
Arrhythmogenic right ventricular cardiomyopathy (ARVC)
Dilated cardiomyopathy
Viral myocarditis
Oxidative phosphorylation
Non-alcoholic fatty liver disease (NAFLD)
Alzheimer's disease
Parkinson's disease
Huntington's disease

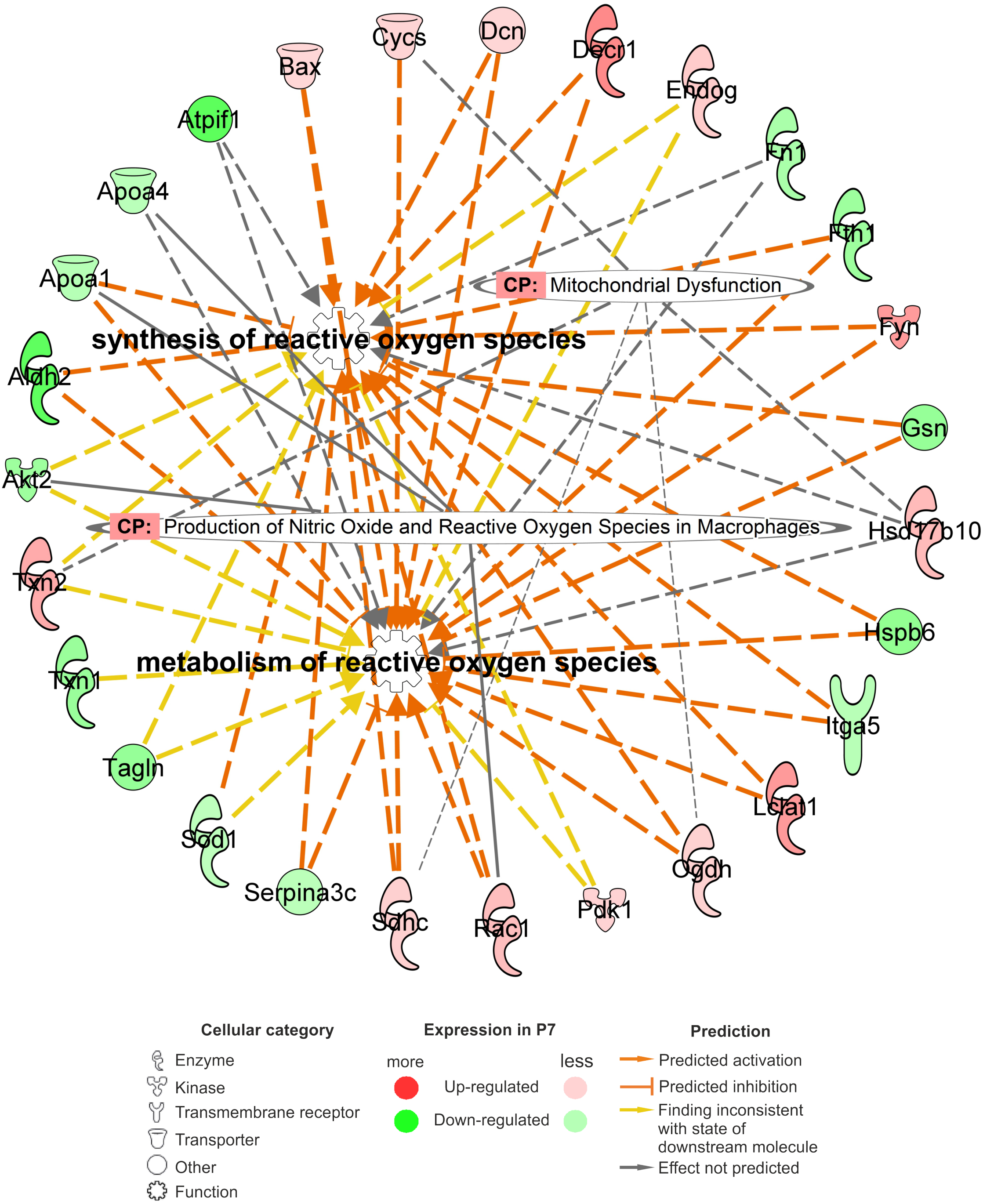
Bax
Cycs
Dcn
Decr1
Endog
Atpif1
Fn1
Apoa4
Fth1
Apoa1
CP: Mitochondrial Dysfunction
Fyn
synthesis of reactive oxygen species
Aldh2
Gsn
Akt2
CP: Production of Nitric Oxide and Reactive Oxygen Species in Macrophages
Hsd17b10
Txn2
Hspb6
metabolism of reactive oxygen species
Txn1
Itga5
Tagln
Lclat1
Sod1
Ogdh
Serpina3c
Sdhc
Rac1
Pdk1
Cellular category
Enzyme
Kinase
Transmembrane receptor
Transporter
Other
Function
Expression in P7
more
less
Up-regulated
Down-regulated
Prediction
Predicted activation
Predicted inhibition
Finding inconsistent with state of downstream molecule
Effect not predicted

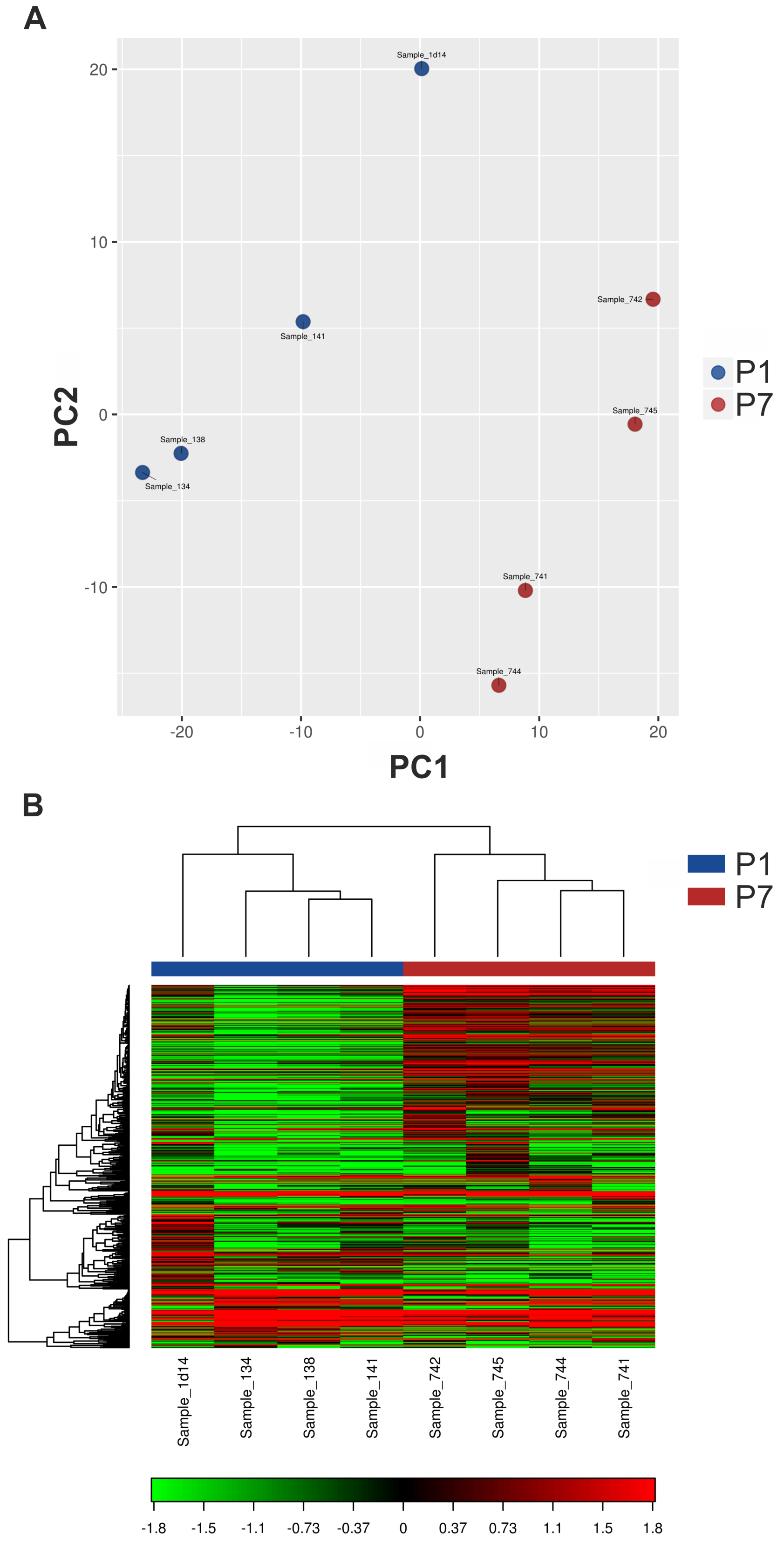
A
PC2
PC1
20
10
0
-10
-20
Sample_1d14
Sample_141
Sample_138
Sample_134
Sample_742
Sample_745
Sample_741
Sample_744
P1
P7
B
P1
P7
Sample_1d14
Sample_134
Sample_138
Sample_141
Sample_742
Sample_745
Sample_744
Sample_741
-1.8
-1.5
-1.1
-0.73
-0.37
0
0.37
0.73
1.1
1.5
1.8

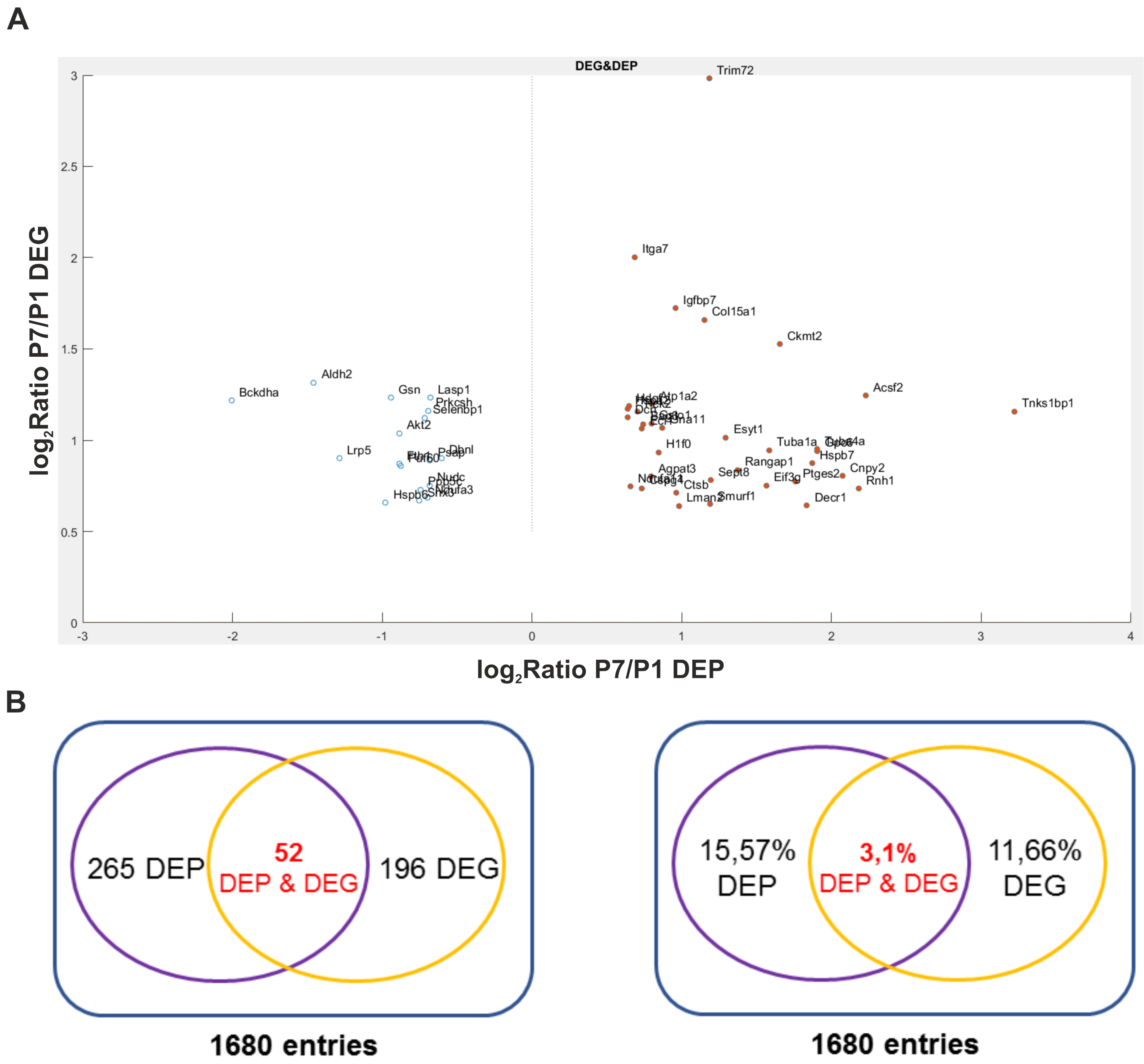
A
DEG&DEP
Trim72
Itga7
Igfbp7
Col15a1
Ckmt2
Acsf2
Tnks1bp1
Esyt1
H1f0
Tuba1a
Hspb7
Rangap1
Agpat3
Sept8
Cnpy2
Ptges2
Eif3g
Rnh1
Ctsb
Lman2
Smurf1
Decr1
Aldh2
Bckdha
Gsn
Lasp1
Prkcsh
Selenbp1
Akt2
Lrp5
Psap
Nudc
Ndufa3
Hspb6
log2Ratio P7/P1 DEG
log2Ratio P7/P1 DEP
B
265 DEP
52
DEP & DEG
196 DEG
1680 entries
15,57%
DEP
3,1%
DEP & DEG
11,66%
DEG
1680 entries

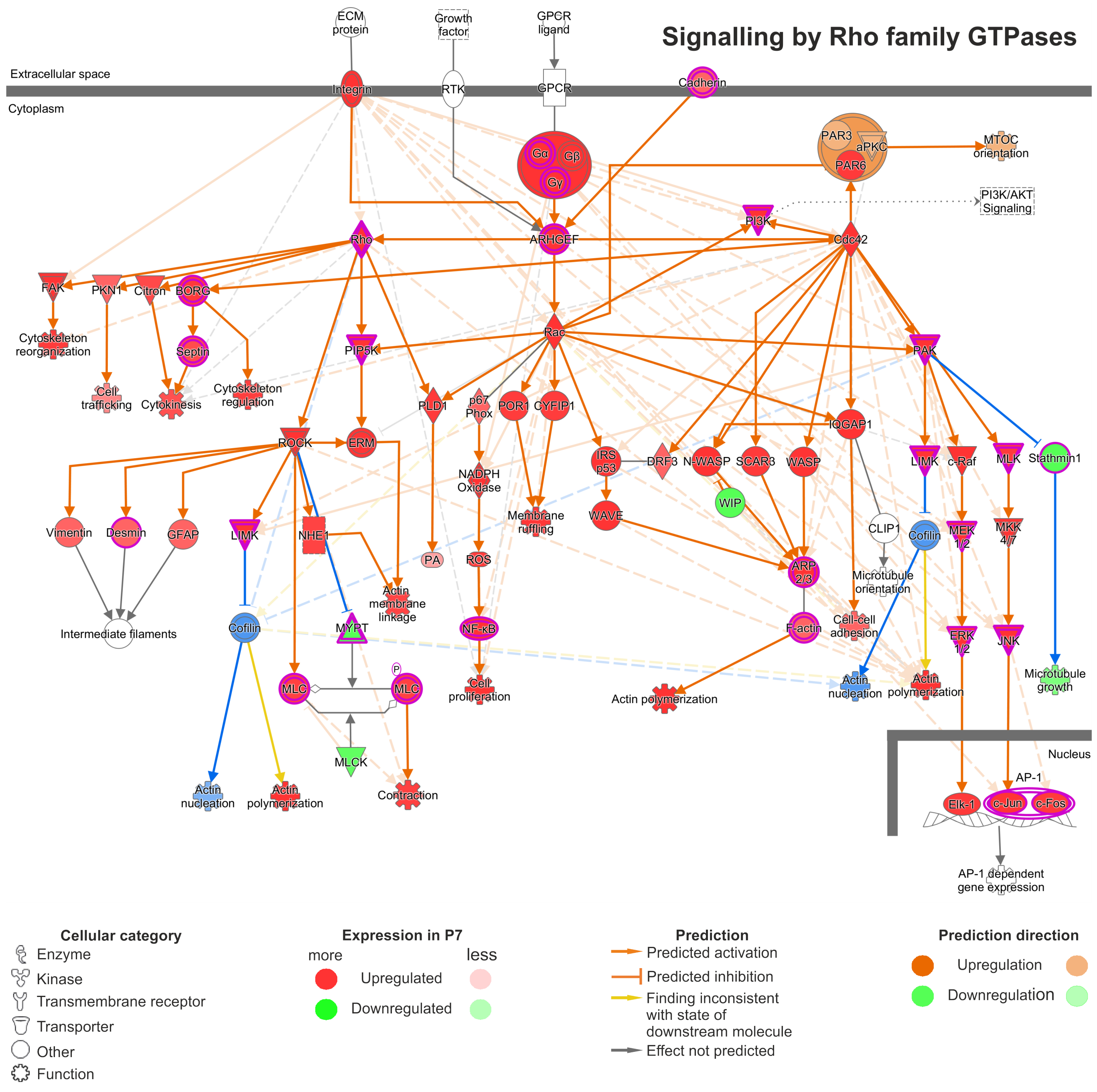
Signalling by Rho family GTPases
ECM protein
Growth factor
GPCR ligand
Extracellular space
Cytoplasm
Integrin
RTK
GPCR
Cadherin
Gα
Gβ
Gγ
PAR3
aPKC
PAR6
MTOC orientation
PI3K/AKT Signaling
PI3K
Rho
ARHGEF
Cdc42
FAK
PKN1
Citron
BORG
Cytoskeleton reorganization
Septin
Cell trafficking
Cytokinesis
Cytoskeleton regulation
Rac
PIP5K
PLD1
p67 Phox
POR1
CYFIP1
IQGAP1
PAK
ROCK
ERM
NADPH Oxidase
Membrane ruffling
IRS p53
DRF3
N-WASP
SCAR3
WASP
LIMK
c-Raf
MLK
Stathmin1
WIP
WAVE
CLIP1
Cofilin
MEK 1/2
MKK 4/7
Vimentin
Desmin
GFAP
LIMK
NHE1
PA
ROS
ARP 2/3
Microtubule orientation
Actin membrane linkage
Intermediate filaments
Cofilin
MYPT
NF-κB
F-actin
Cell-cell adhesion
ERK 1/2
JNK
Actin nucleation
Actin polymerization
Microtubule growth
P
MLC
MLC
Cell proliferation
Actin polymerization
MLCK
Nucleus
AP-1
Elk-1
c-Jun
c-Fos
Actin nucleation
Actin polymerization
Contraction
AP-1 dependent gene expression
Cellular category
Enzyme
Kinase
Transmembrane receptor
Transporter
Other
Function
Expression in P7
more
less
Upregulated
Downregulated
Prediction
Predicted activation
Predicted inhibition
Finding inconsistent with state of downstream molecule
Effect not predicted
Prediction direction
Upregulation
Downregulation

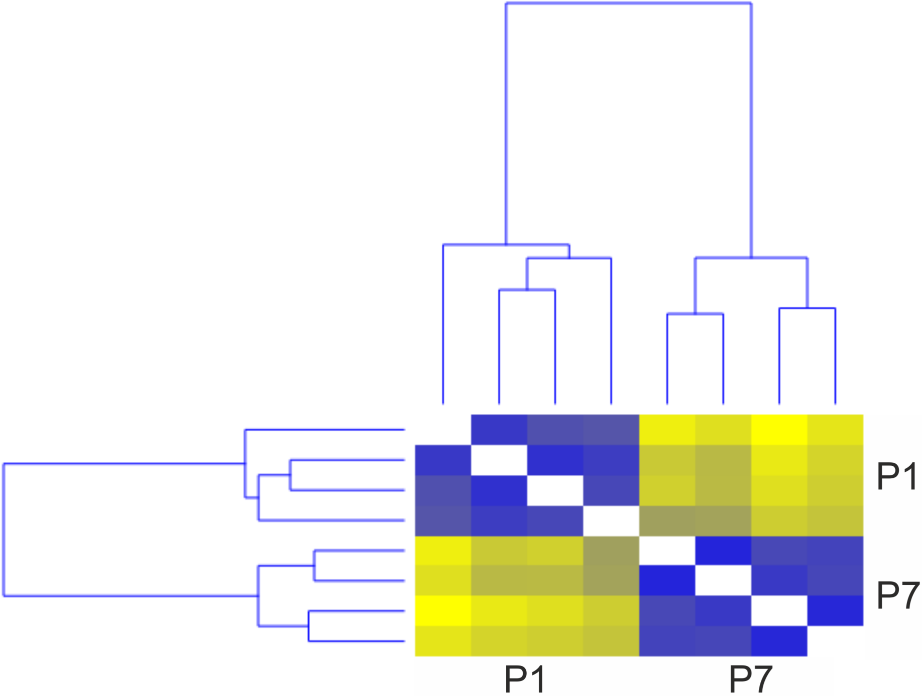

P1
P7
P1
P7

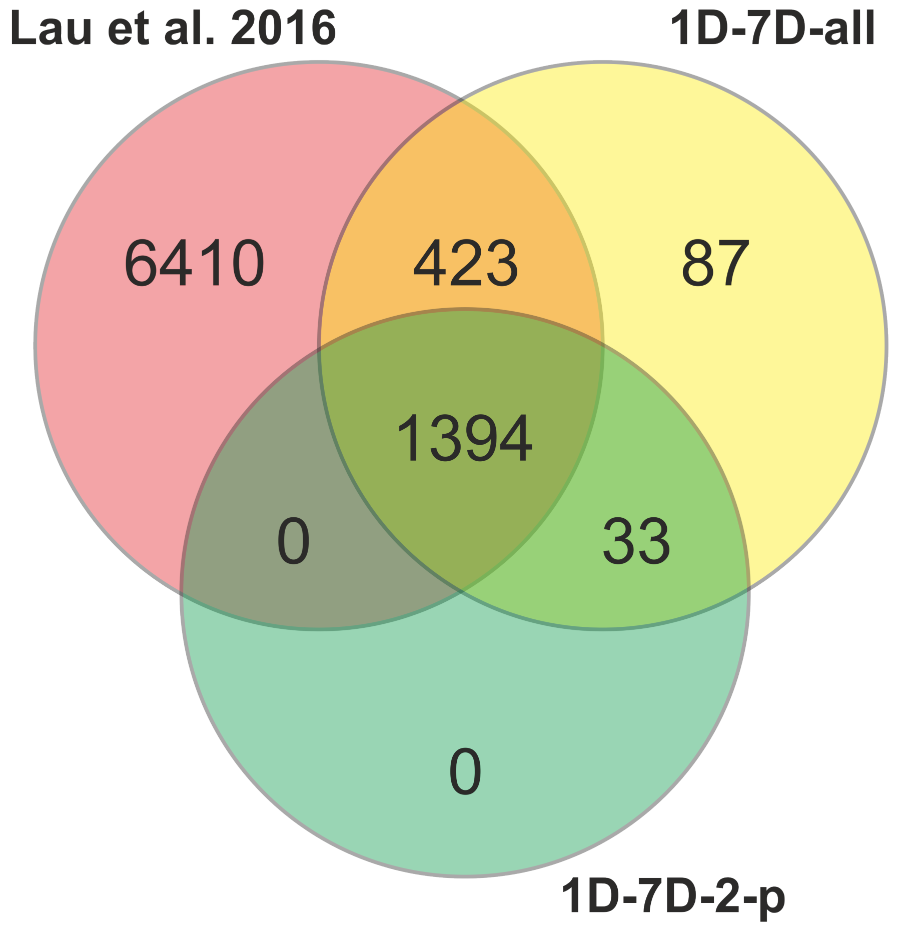
Lau et al. 2016
1D-7D-all
6410
423
87
1394
0
33
0
1D-7D-2-p
